# Gammaproteobacteria, a core taxon in the guts of soil fauna, are potential responders to environmental concentrations of soil pollutants

**DOI:** 10.1186/s40168-021-01150-6

**Published:** 2021-09-30

**Authors:** Qi Zhang, Zhenyan Zhang, Tao Lu, Yitian Yu, Josep Penuelas, Yong-Guan Zhu, Haifeng Qian

**Affiliations:** 1 College of Environment, Zhejiang University of Technology, Hangzhou, 310032 P. R. China; 2 Global Ecology Unit CREAF-CSIC-UAB, CSIC, Bellaterra, 08193 Barcelona, Catalonia Spain; 3 CREAF, Campus Universitat Autònoma de Barcelona, Cerdanyola del Vallès, 08193 Barcelona, Catalonia Spain; 4 Key Laboratory of Urban Environment and Health, Institute of Urban Environment, Chinese Academy of Sciences, Xiamen, 361021 P. R. China; 5 State Key Lab of Urban and Regional Ecology, Research Center for Ecoenvironmental Sciences, Chinese Academy of Sciences, Beijing, 100085 P. R. China

**Keywords:** Gut microbiota, Pesticide, Indicator taxa, Machine learning, Soil invertebrate, Antibiotic resistance genes

## Abstract

**Background:**

The ubiquitous gut microbiotas acquired from the environment contribute to host health. The gut microbiotas of soil invertebrates are gradually assembled from the microecological region of the soil ecosystem which they inhabit, but little is known about their characteristics when the hosts are under environmental stress. The rapid development of high-throughput DNA sequencing in the last decade has provided unprecedented insights and opportunities to characterize the gut microbiotas of soil invertebrates. Here, we characterized the core, transient, and rare bacterial taxa in the guts of soil invertebrates using the core index (CI) and developed a new theory of global microbial diversity of soil ecological microregions.

**Results:**

We found that the Gammaproteobacteria could respond indiscriminately to the exposure to environmental concentrations of soil pollutants and were closely associated with the physiology and function of the host. Meanwhile, machine-learning models based on metadata calculated that Gammaproteobacteria were the core bacteria with the highest colonization potential in the gut, and further identified that they were the best indicator taxon of the response to environmental concentrations of soil pollution. Gammaproteobacteria also closely correlated with the abundance of antibiotic resistance genes.

**Conclusions:**

Our results determined that Gammaproteobacteria were an indicator taxon in the guts of the soil invertebrates that responded to environmental concentrations of soil pollutants, thus providing an effective theoretical basis for subsequent assessments of soil ecological risk. The results of the physiological and biochemical analyses of the host and the microbial-community functions, and the antibiotic resistance of Gammaproteobacteria, provide new insights for evaluating global soil ecological health.

**Video abstract**

**Supplementary Information:**

The online version contains supplementary material available at 10.1186/s40168-021-01150-6.

## Background

Gut microbiotas help to maintain the health and physiological homeostasis of metazoans, in which epithelial membranes are the most important interfaces between an organism and its environment [1]. In particular, symbiotic gut microbes promote the digestion of complex substrates and reduce intestinal colonization by pathogens through training of the host immune system and by affecting body maturation [2]. The complexity of mammalian gut microbiotas hinders the analysis of host-microbiota interactions, so invertebrates harboring simple gut microbiotas are an important experimental model for determining the relative contributions of individual microbes to the physiological processes of the host [3].

Soil fauna account for 23% of known animals and are essential components of soil ecosystems, involved in the decomposition of litter, the transfer of energy, and the formation of soil microstructure [4, 5]. Recent studies have found that the gut microbiotas of soil invertebrates are important for microbial research and have been associated with the enrichment and dissemination of genes conferring antibiotic resistance [6], the colonization and transfer of pathogens [7], and the potential for the cycling of soil elements [8], thereby expanding the ecological and evolutionary potential of the hosts [9]. The gut tract of soil invertebrates is dominated by transient microbiota [5], depending on time, environmental conditions, nutrient availability, stage of growth and health of the host, and even circadian rhythms [10]. Therefore, characterizing the core gut microbiotas of soil invertebrates is challenging.

Soil pollution is one of the most serious hidden dangers to global agricultural food security [11]. Soil invertebrate faunae, as some of the first organisms exposed to soil pollutants, are often used as indicator species in toxicological experiments [12, 13]. The residual environmental concentrations of soil pollutants are usually low and rarely greatly affect the physiology of soil invertebrate fauna in the short term. The microbiotas within the guts of soil invertebrates have more sensitive characteristics than their hosts or even soil microbiotas [14, 15]. The process to identify core microbiota is inevitably affected by different experimental methods, PCR primers, sequencing depth, and other technical operations [16]. Thus, core microbiota that can stably exist within the gut of a species have the potential to become an indicator organism for general study. The development of a single test, however, is labor intensive and would not be universal, so identifying a general indicator taxon is difficult.

A method combining metadata analysis and machine learning has recently been used to predict the development of cancer based on human intestinal microbiotas [17], predict the occurrence of fusarium wilt in soil based on soil microbiotas [18], and predict microplastic type and its disposal methods based on characteristic environmental microbiotas [19]. The emergence of research interest in the gut microbiotas of soil fauna, the substantial development of sequencing technology, the advocacy of publicly available data, and machine learning, together, provide an effective route to obtain and merge the considerable public sequencing data related to the gut microbiotas of soil fauna, for the identification of not only the core gut microbiotas of soil fauna but also of indicator species that respond to soil pollutants.

Oxytetracycline (OTC) is the most commonly used antibiotic in animal husbandry and several studies indicated that OTC is a common residual antibiotic in soil to which manure has been applied [20, 21]. The strobilurin fungicide azoxystrobin (AZ) is the highest selling fungicide, with $1.165 billion in global sales in 2016 (http://cn.agropages.com/, accessed on October 19, 2017). Both the antibiotic OTC and the fungicide AZ are broad-spectrum antimicrobials, and are the most representative of soil antimicrobials. Therefore, we used AZ and OTC as typical treatments to investigate the changes of the gut microbiota in the model soil invertebrate *Folsomia candida* (Collembola) and analyzed functional genes using eukaryotic transcriptome sequencing, which could identify the interactions between the gut microbiota and host physiological functions. We then defined the core gut microbiota of *F. candida* using a large-scale analysis of data for the microbial community and identified taxa that could be used as potential indicators of environmental concentrations of soil pollutants using random forest machine learning. We further investigated the antibiotic resistance genes (ARGs) in *F. candida* gut using HT-qPCR and identified the taxa related to the ARGs.

## Materials and methods

Details are provided in *Supplementary Information* and Figure S1.

### Laboratory experiments

#### Experimental design

The test species, *Folsomia candida* (“Berlin strain,” originally obtained from Aarhus University, Denmark), was reared in our laboratory for 2 years following Organization for Economic Co-operation and Development (OECD) guideline 232 and was placed in a suitable breeding environment, maintained in Petri dishes containing a mixture of charcoal and plaster of Paris (1:8 w/w). These Petri dishes were kept in a thermostatic box (Safe Co., Ningbo, China) at 75% relative humidity and a temperature of 20 ± 2 °C with a 16:8-h light: dark photoperiod (800 Lux). Ultrapure water was added once a week to ensure substrate moisture and the *F. candida* were fed twice a week with dry yeast powder.

To obtain *F. candida*, all of the same age, we transferred 60-70 active adults to a new substrate to lay eggs for 2 days, and then removed these adults. As the juveniles hatched, they were immediately transferred to a new substrate for culture. Before the exposure experiment, 7-9-day-old collembolans were transferred to the test soil (2.27, 16.47, and 81.25% of clay, silt, and sand, respectively; pH 5.16; water-holding capacity, 46.8%; total N content, 3.8 g/kg) from a vegetable field (29° 49′ N, 121° 20′ E; Zhejiang, China) to adapt the test collembolans to the new culture environment.

When the mortality of these collembolans in the new soil culture environment was less than 1% during the pre-incubation process, we used these collembolans for subsequent exposure experiments. To simulate the natural field environment, we did not feed in the pre-incubation and exposure experiments and added distilled water twice weekly to maintain soil moisture.

We firstly conducted a pre-exposure experiment, to measure reproduction rate and mortality, for selecting the most suitable concentration for the formal experiments, and the specific details are described in the *Supplementary Information*. The formal laboratory experiments were divided into two parts, one for determining the death, reproduction, locomotion, reactive oxygen species (ROS) concentration, and cytochrome P450 (CYP450) enzymatic activity of *F. candida*, and the other for transcriptomic measurements (gene expression), the gut microbiota (bacterial and fungal communities), and the gut resistome of *F. candida*. Before starting the experiments, we added ultrapure water to restore the microorganisms in the soil for a week at room temperature, and the moisture content was maintained at 60% of the maximum (60.23%), as detailed in OECD guideline 232. The laboratory experiments were divided into two parts, one for determining the death, reproduction, locomotion, ROS concentration, and CYP450 enzymatic activity of *F. candida*, and the other for transcriptomic measurements (gene expression), the gut microbiota (bacterial and fungal communities), and gut resistome of *F. candida*. In one part of the experiment, soil microcosms were established for exposing *F. candida* to the pollutants: control (no pollutants), AZ (0.3 mg AZ/kg dry soil), OTC (10 mg/kg dry soil), and AO (combined 0.3 mg AZ/kg dry soil and 10 mg OTC/kg dry soil). Twenty 14-16-day-old pre-incubated collembolans were introduced into these experimental microcosms, each treatment had four replicates and were conducted in sterile glass beakers (inner diameter 5.3 cm, 6.5 cm high) containing 30 g of moist soil at 18 °C with a diurnal light cycle (16:8 h light: dark). We added sterile water twice a week and recorded the numbers of adults and larvae in all microcosms after 28 days of exposure using the imageJ 1.52, and 13 adults were collected for the locomotory test. Two adults were then isolated for determining ROS content and CYP450 enzymatic activity respectively, following the manufacturer’s instructions for the nematode ELISA kit based on the double-antibody sandwich method (Jiangsu Enzyme Industrial Co., Ltd., Yancheng, China) [15].

In the other experimental setup, sixty 14-16-day-old pre-incubated collembolans were added to control, AZ, OTC, and AO experimental microcosms with eight replicates. These experimental microcosms were conducted in same-sized beaker with 65 g moist soil. After exposure for 28 days, all collembolans were collected for gut microbiome, resistome, and eukaryotic transcriptome analysis. Fifty adults per replicate were washed three times with ultrapure water and dissected under a stereo microscope using very precise tweezers to obtain the collembolan gut samples [6].

The gut samples were transferred to 2-mL round-bottomed centrifuge tubes containing 20 μL of proteinase K and 180 μL of a lysis buffer solution for the extraction of DNA [15]. A total of 2 g of soil per sample, without collembolans, was collected for analyzing the soil microbial community. Fifty adults each replicate were provided for extraction of RNA. These collected collembolans were firstly washed three times with ultrapure water and then immediately frozen in liquid nitrogen for RNA extraction.

#### Effects of soil pollution on *F. candida* gut microbiota

A total of 50 guts of adult *F. candida*, per replicate, were used to isolate high-quality DNA using a DNeasy® Blood & Tissue Kit (QIAGEN, GER). The V4 hypervariable region of the 16S rRNA gene was amplified using universal primers (forward primer 515F 5′-GTGCCAGCMGCCGCGG-3′ and reverse primer 806R 5′-GGACTACNVGGGTWTCTAA-3′) [14], and region 1 of the internal transcribed spacer (ITS) gene was amplified using the forward primer ITS1F (5′-CTTGGTCATTTAGAGGAAGTAA-3′) and the reverse primer ITS2 (5′-GCTGCGTTCTTCATCGATGC-3′) [21]. The 50-μL reactions (25 μL of TaKaRa ExTaq DNA polymerase, 1 μL of DNA (range 10-15 μg/mL), 1 μL of universal forward, 1 μL reverse primer, and 22 μL of PCR-grade water) were amplified following reaction conditions previously described [14]. The PCR products were then purified, pooled, and sequenced using the Illumina MiseqPE300 platform (Meiji, Shanghai, China).

#### Effects of soil pollution on *F. candida* gut resistome

We used a total of 384 primer sets (*Data set S**2*) (including 320 antibiotic resistance genes (ARGs), 57 mobile genetic elements (MGEs), and the 16S rRNA gene) to investigate the composition and abundance of ARGs in the collembolan gut using the SmartChip Real-time PCR system (Wafergen, USA). The PCR reaction mixture of each well compose with collembolan DNA template, primers, sterile water, and LightCycler 480 SYBR Green I Master mix. The reaction system (95 °C (10 min) and 40 cycles of 95 °C (0.5 min) and 60 °C (0.5 min)) was used for HT qPCR. SmartChip qPCR software was used to analyze the raw data and a threshold cycle (CT) of 31 was used to detect ARGs. Only when 3 technical replicates and 3 biological replicates were amplified at the same time, did we consider an ARG to have been detected. The relative abundance of ARGs was calculated using the equation below [22].

*Copy number of gene* = 10 ^((31-*CT* (measurement))/(10/3)^

#### Effects of soil pollution on *F. candida* gene expression at the transcriptomic level

A total of 50 collected adults per replicate were immediately snap frozen in liquid nitrogen and stored at −80 °C to ensure the RNA integrity. The RNA was isolated from each replicate pool using an RNA extraction kit (HiPure Universal RNA Midi Kit, Magen, Guangzhou, China). We checked the concentration, purity, and integrity of the RNA and isolated the mRNA. The enriched mRNA was then randomly broken into fragments of ~300 bp and reverse transcribed to produce stable double-stranded cDNA using ReverTra Ace qPCR RT Kit (TOYOBO, Osaka, Japan), which was sequenced using the Illumina Novaseq 6000 platform (Meiji, Shanghai, China).

#### Data collection and description and processing of the 16S rRNA gene metadata

Based on a previous search principle [19], we searched the Web of Science Core Collection and Science Direct for the terms “gut microbiota of soil fauna or ‘species’ name” “gut microbial community of soil fauna or ‘species’ name” and “gut microbiota of soil invertebrate or ‘species’ name.” A total of 33 studies were collected from these databases, but only 20 independent experiments were publicly available with incomplete 16S rRNA gene sequences (*Data set S**1*), including three 16S rRNA gene hypervariable regions, V4, V4-V5, and V3-V4, which were amplified using the respective primer pairs 515F/806R (forward primer: 5′-GTGCCAGCMGCCGCGGTAA-3′, reverse primer: 5′-GGACTACNVGGGTWTCTAA-3′), 515F/907R (forward primer: 5′-GTGCCAGCMGCCGCGGTAA-3′, reverse primer: 5′-CCGTCAATTCMTTTRAGTTT-3′), and 334F/806R (forward primer: 5′-CCTACGGGAGGCAGCAG-3′, reverse primer: 5′-GGACTACHVGGGTWTCTAAT-3′). We used Vsearch (version 2.7.1) and QIIME2 (version 2021.2) to filter, classify, and merge these raw data sets. Each sample sequence was normalized to 2000 reads, and a total of 415 samples from the 17 independent studies, including the 17 soil pollutants (e.g., the fungicide azoxystrobin; the insecticide cypermethrin; the herbicide glyphosate; the antibiotics tetracycline, sulfamethoxazole, and oxytetracycline; the antibiotic substitute *Macleaya cordata* extract; the heavy metals arsenic, silver nitrate, silver nanoparticles, and nano-copper oxide; and the emerging pollutants micro-, nano-, and tire-tread plastics), five kinds of soil invertebrates (collembolans, enchytraeus, earthworms, mites, and ants), and three exposure methods (oral exposure, soil microcosm, and field experiment). All metadata were divided into “Control” and “Pollution” based on the sample information for each experiment (*Data set S**1*).

#### Construction and validation of the predictive model

Machine-learning algorithms were used to predict the heterogeneous microbial data for identifying taxa in the guts of the soil invertebrates that were most strongly associated with the stress of the soil pollutants. We utilized three machine-learning algorithms, random forest (RF) [23], logistic regression (LR) [24], and support-vector machine (SVM) [25]. The receiver operating characteristic curve (ROC) and the area under the curve indicated that the RF algorithm performed well on our merged data. We therefore used the RF algorithm to build the predictive model. Classification models based on each taxonomic level could distinguish between the bacterial communities in the guts of the soil invertebrates in the control and pollutant treatments using the randomForest package in R with default parameters (version 4.6-14). The results indicated that the average accuracy rate was similar across all taxa, so we selected the RF model at the genus level with the lowest estimated rate of out-of-bag (OOB) errors (17.58%) [26].

### Statistical methods

The means ± standard errors (SEs) of each treatment were calculated. A two-tailed Welch’s *t* test was used to identify significant differences between groups. A principal coordinate analysis (PCoA) based on unweighted UniFrac distances for the guts of the soil invertebrates and for the bacterial and fungal communities in the surrounding soil was performed using the Majorbio Cloud Platform (www.majorbio.com), and the output was visualized using OriginPro 9.1. The Adonis function (9999 permutations) was used in a PERMANOVA to identify differences among the treatments using the vegan 2.4-3 package in R version 3.6.1. Function prediction analysis of fungi using FUNGuild was performed on Majorbio Cloud Platform (www.majorbio.com).

Heatmaps were generated using TBtools (Toolbox Biologists v0.655), and histograms, line and box charts, and linear regressions were produced using GraphPad Prism 8.00. The weighted gene co-expression network analysis (WGCNA) was performed using the Majorbio Cloud Platform (www.majorbio.com). The co-occurrence network analysis of laboratory experiments, based on the relative abundance of all bacterial and fungal classes, using pairwise Spearman’s rank correlations (*r*) in the psych package in R (*r* > 0.6 or *r* < −0.6, *P* < 0.05), was performed using Gephi v0.9.2. The shared network based on the frequency of each bacterial class among all independent studies was produced using Gephi v0.9.2. Structural equation models (SEMs) were built to calculate the direct and indirect effects among the gut bacteria (Shannon index), treated groups, bacteria_Shannon_/fungi_Shannon_ (B/F) index, cytochrome P450 (enzymatic activity), the HAA index, Gammaproteobacteria (relative abundance), and transcriptome (PC1 of the TPM value using Bray-Curtis distances). The significance of each path-coefficient was analyzed by calculating its critical ratio (*P* < 0.05). The goodness-of-fit index (GFI) and the Bentler comparative fit index (CFI) indicating the goodness-of-fit of the models to the original data. The SEM was produced using Amos Graphics v22 (IBM Corp., Armonk, NY, USA). Meta-analysis and sensitivity analysis were performed using the STATA statistical software package version 15.0 (Stata Corp., College Station, TX, USA). In addition, to characterize positive and negative co-occurrences separately, we used the cohesion among taxa to reveal the interactions, similarity, and differences between both positive and negative species interactions in the niches of microbial taxa, using the previous equation to calculate the positive and negative cohesion values [27].

## Results

### Effects of AZ, OTC, and AO on the physiology and biochemistry of *F. candida*.

**Fig. 1:**
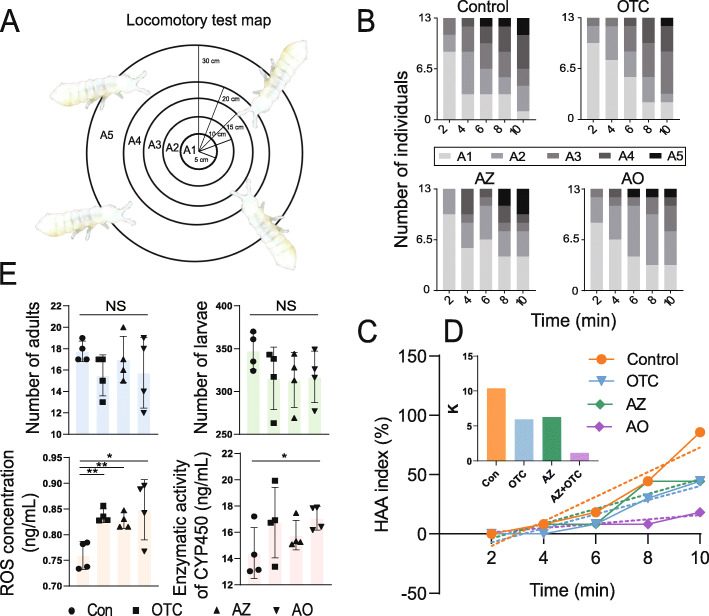
Effects of azoxystrobin (AZ), oxytetracycline (OTC), and their combination (AO) on the physiology and biochemistry of *Folsomia candida*. Locomotory test map (**A**). The number of the individuals in different areas of the map after 2-10 min of exposure to AZ, OTC, and AO (**B**). The high area activity (HAA) index (**C**), with different *K* values (**D**). **E** The numbers of adults and larvae, ROS concentrations, and the enzymatic activity of CYP450 after exposure to AZ, OTC, and AO. * (*P* < 0.05) and ** (*P* < 0.01) indicate significant differences between the bacterial and fungal communities (two-tailed Welch’s *t* test)

We designed a locomotory map (radii of 5, 10, 15, 20, and 30 cm) to characterize the motility of *F. candida* after exposure to pollutants (Fig. 1A). Thirteen individuals of the same size were treated with 0.3 mg AZ/kg dry soil, 10 mg OTC/kg dry soil, or AO for 28 days and were then put at the center of the map. The number of individuals in areas A1-A5 after 2, 4, 6, 8, and 10 min were recorded (Fig. 1B). The AZ and OTC concentrations, which did not cause mortality, were chosen according to the environmental residual levels and pre-experiment results (Figure S2). *F. candida* motility was calculated using Eq. 1 (*Supplementary Information*), and an index of high area activity (HAA) was calculated for each time point. *F. candida* motility for the treatments was in the order control > AZ > TOC > AO based on the slopes of the fitted lines (Fig. 1C, D).

The numbers of adults and larvae did not differ significantly between the treated groups and the control. The concentration of reactive oxygen species (ROS) was higher in OTC-, AZ-, and AO-treated groups than the control (Fig. 1E: *P* < 0.01, *P* < 0.01, *P* < 0.05, *df* = 6, *n* = 4 per group, two-tailed Welch’s *t* test), and the enzymatic activity of cytochrome P450 (CYP450) was significantly higher only in the AO group (*P* < 0.05, *df* = 6, *n* = 4 per group, two-tailed Welch’s *t* test).

### Effects of AZ, OTC, and AO on the microbiota of the surrounding soil.

A total of 0.5 g of soil surrounding the *F. candida* specimens was carefully separated from the microcosms, both with and without treatment, under a microscope to avoid interference by the larvae for determining the effects of AZ, OTC, and AO on the diversity and composition of the soil microbiota. The soil bacterial and fungal communities were assessed using deep amplicon sequencing of the 16S rRNA and internal transcribed spacer (ITS) genes, respectively. Exposure to AZ, OTC, and AO for 28 days did not significantly affect the alpha- (Shannon and Chao indices) or beta-diversity of the soil bacterial community (*P* > 0.05, *df* = 6, *n* = 4 per group, two-tailed Welch’s *t* test; PERMANOVA for treatments: *R*^2^ = 0.2764, *P* = 0.182, Adonis analysis) (Figure S3A, B). The number and classification of operational taxonomic units (OTUs) did not differ significantly between the treated groups and the control (*P* > 0.05, *df* = 6, *n* = 4 per group, metagenomeSeq difference analysis).

Fungal diversity was significantly higher in the AZ and AO groups, although the fungi were richest in the AO group (Figure S3C), and the treatments affected the composition of the fungal community (principal coordinate analysis (PCoA) of unweighted UniFrac distance) [28], PERMANOVA for treatments: *R*^2^ = 0.2392, *P* = 0.024, Adonis analysis) (Figure S3D). The number and classification of OTUs differed significantly in each group (Figure S4A-C), and the OTUs with highest abundances belonged to plant or animal pathogens (function prediction analysis of fungi using FUNGuild, a tool for parsing fungal OTUs) (Figure S4D). This finding indicated that soil microbiotas would respond differently to different soil pollutants and that low environmental concentrations of pollutants would enrich potential soil pathogens, inducing ecological risks.

### Effects of AZ, OTC, and AO on gut microbiotas of *F. candida*

Analysis of the 16S rRNA and ITS gene sequences from 20 adult *F. candida* guts showed that richness was significantly higher in the fungal community than the bacterial community (*P* < 0.001, *df* = 30, two-tailed Welch’s *t* test) (Figure S5), indicating that the potential colonization in *F. candida* guts was much lower for fungi than bacteria. The addition of pollutants did not affect the diversity or abundance of the fungal community (Shannon and Chao indices: *P* > 0.05, *df* = 6, two-tailed Welch’s *t* test) (Figure S6A, B), but unweighted UniFrac distances indicated that the diversity and abundance of the fungal community differed between the AO group and the control (*P* <0.001, *df* = 26, two-tailed Welch’s *t* test) (Figure S6C), perhaps due to the interaction between fungi and bacteria. We established a co-occurrence network of the bacterial and fungal species to test this hypothesis and found that the index of stability (*negative*: *positive* cohesion) for all microbial communities was highest in the AO group, representing the most unstable community network (Figure S7).

**Fig. 2:**
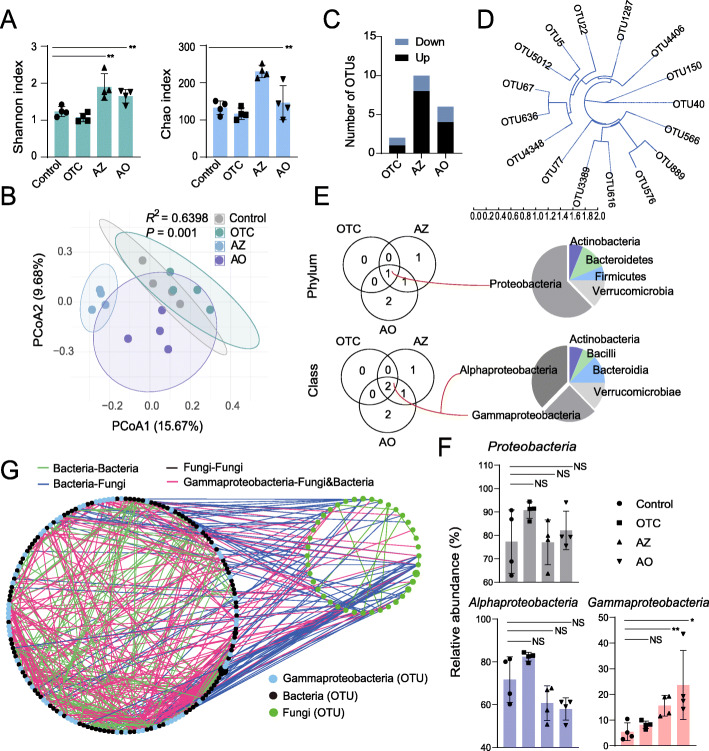
Effects of azoxystrobin (AZ), oxytetracycline (OTC), and their combination (AO) on the gut microbiota of *Folsomia candida*. Alpha diversity (Shannon and Chao indices) (**A**). PCoA of OTU file data using unweighted UniFrac distances) for different groups of gut bacteria (**B**). Significantly different OTUs between the control and treated groups (**C**). Evolutionary relationships of the OTUs (**D**). Classification of significantly different OTUs at the phylum and class levels and significantly different OTUs shared between the treated groups (**E**). The relative abundance of significantly different OTUs shared in all treated groups (**F**). Co-occurrence network analysis identifing the relationships among the bacteria, fungi, and Gammaproteobacteria (**G**). The size and color of the nodes represent the relative abundance and the associated type of OTU data (black, bacteria; green, fungi; and blue, Gammaproteobacteria). The colored lines indicate the links among the bacteria, fungi, and Gammaproteobacteria. * (*P* < 0.05) and ** (*P* < 0.01) indicate significant differences between the bacterial and fungal communities (two-tailed Welch’s *t* test), NS indicates “not significant”

Exposure to the pollutants significantly affected the diversity and structure of the bacterial community, especially in the AZ and AO groups (Fig. 2A, B: AZ_Shannon_ and AZ_Chao_: *P* < 0.01, *df* = 6, two-tailed Welch’s *t* test; AO_Shannon_: *P* < 0.01, *df* = 6, two-tailed Welch’s *t* test; PERMANOVA for treatments: *R*^2^ = 0.6398, *P* = 0.001, Adonis analysis). Totals of 2 (1 up and 1 down), 10 (8 up and 2 down), and 6 (4 up and 2 down) OTUs differed significantly between the OTC, AZ, and AO groups as compared to the control, respectively (Fig. 2C: *P* < 0.05, *df* = 6, *n* = 4 per group, MetagenomeSeq difference analysis). The associated phylogenetic relationships are shown in Fig. 2D. The OTUs shared between the treatment groups were classified into Proteobacteria (phylum), with Alphaproteobacteria and Gammaproteobacteria (classes) identified as common potential indicator taxa after exposure to the pollutants (Fig. 2E), similar to previous studies [22, 29]. Proteobacteria may be a potential microbial signature of dysbiosis in soil invertebrates, just as in human guts [30]. Interestingly, the relative abundance of Gammaproteobacteria gradually increased with the intensity of pollutant stress (OTC < AZ < AO) (Fig. 2F: *P* > 0.05, *P* < 0.01, and *P* < 0.05, *df* = 6, *n* = 4 per group, two-tailed Welch’s *t* test). The co-occurrence network indicated that the Gammaproteobacteria, the most common taxon across all treatments, was significantly correlated with the abundances of 178 other bacteria and 16 fungi, indicating that this taxon occupied an important central position for correlating the fungal and bacterial communities (Fig. 2G, Spearman’s ⍴ > 0.6, *P* < 0.05). The associated topological data also showed that Gammaproteobacteria played the most important role in the co-occurrence network through interactions with other bacteria and fungi (Figure S8).

### Relationships between Gammaproteobacteria and host physiology, biochemistry, and function

**Fig. 3:**
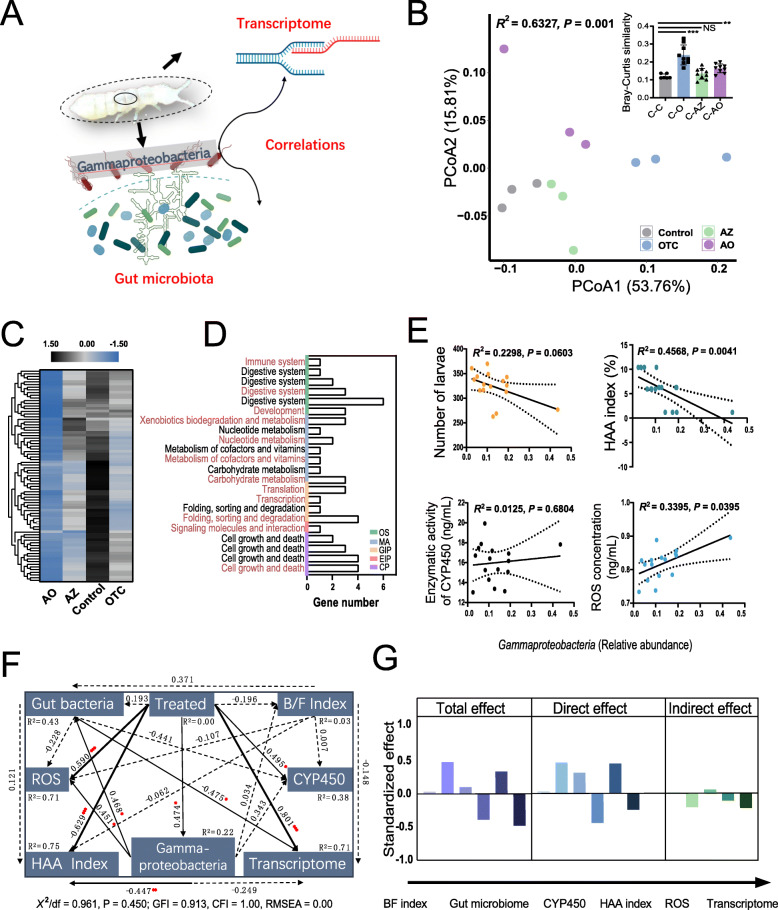
Correlations among the transcriptome, gut microbiome, and gut Gammaproteobacteria of *Folsomia candida* (A). PCoA based on the *Folsomia candida* gene expression value (TPM) data using Bray-Curtis distances showing the different clusters in the control and the treated groups (OTC, AZ, and AO) (**B**). Heatmap of the level of expression (the color key indicates the TPM value) (**C**), and associated KEGG functional pathway (the color scale and red text indicate the primary and various secondary pathways, respectively) (**D**). Ordinary least squares (OLS) linear regression between Gammaproteobacteria (relative abundance) and physiological and biochemical indicators of *F. candida* (number of juveniles, HAA index, enzymatic activity of CYP450, and ROS concentration) (**E**). Structural equation model (SEM) of the relationships among the gut bacteria (Shannon index), treated groups, bacteria_Shannon_/fungi_Shannon_ (B/F) index, cytochrome P450 (enzymatic activity), the HAA index, Gammaproteobacteria (relative abundance), transcriptome (PC1 of the TPM value using Bray-Curtis distances), and the goodness-of-fit index (GFI) and the Bentler comparative fit index (CFI) indicating the goodness-of-fit of the models to the original data. Dashed lines indicates the “not significant correlation” (**F**). The direct, indirect, and total standardized effects of Gammaproteobacteria on the indicators (**G**)

We collected 50 *F. candida* after 28 days of exposure to AZ, OTC, and AO to extract RNA for transcriptome sequencing based on the *F. candida* genome in NCBI (GCF_002217175.1) [31] after transferring the gut contents to sterile water to avoid contamination from gut microorganisms (Fig. 3A). A PCoA of gene expression value (TPM) using the Bray-Curtis distances indicated differences among the groups (Fig. 3B: PERMANOVA for treatments: *R*^2^ = 0.6327, *P* = 0.001), and Bray-Curtis dissimilarity indicated that the OTC and AO groups were significantly separated from the control (*P* < 0.001 and *P* < 0.01, respectively, two-tailed Welch’s *t* test). We classified a total of 24,436 genes (21,359 known, 3077 unknown) into five unique module eigengenes based on differences in the strength of the interaction between genes, conforming to a scale-free distribution, to determine whether gut Gammaproteobacteria were associated with host functions (*R*^2^ = 0.82, soft power (*β*) = 6, minimum module size = 30, weighted gene co-expression network (WGCNA) analysis) (Figure S9A-C). The MEbrown (gene membership of brown) co-expression module was selected for further analysis, because it was most relevant to the control phenotype, to distinguish between the control and treated groups (module significance = 0.451, module membership vs gene significant: *R*^2^ = −0.697, *P* < 0.012, WGCNA analysis) (Figure S9D-F). Seventy-nine MEbrown genes were significantly negatively correlated with the relative abundance of Gammaproteobacteria (Spearman’s ⍴ > 0.6, *P* < 0.05, Spearman analysis), and gene expression was lower in the treated groups than the control (Fig. 3C). These genes were annotated to functions of immunity, digestion, development, detoxification, vitamin carbohydrate metabolism, protein assembly and synthesis, and cell growth and apoptosis based on the KEGG pathway database (Fig. 3D). Locomotive activity (HAA index) and ROS concentration for the host were also significantly correlated with the relative abundance of Gammaproteobacteria (Fig. 3E: *R*^2^ = −0.4568, *P* = 0.0041; *R*^2^ = 0.3395, *P* = 0.0395, ordinary least squares linear regression analysis). These findings indicated that the relative abundance of gut Gammaproteobacteria was negatively correlated with the normal physiological functions of the host, similar to the index of oxidative stress (ROS level), indicating that the host was stressed. Structural equation modeling (SEM) found that the gut Gammaproteobacteria were connected with the expression of host functional genes by interfering with the gut bacterial community and were also directly associated with host locomotion and level of oxidative stress (Fig. 3F, G: *χ*^2^/*df* = 0.961, *P* = 0.450; GFI = 0.913, CFI = 1.00, RAMSEA = 0.000, SEM analysis).

### Defining the core microbiotas of the guts of soil invertebrates based on a metadata analysis

Global databases of soil invertebrates have recently been developed, but are concentrated mainly on species identification (Global Biodiversity Information Facility (GBIF), www.gbif.org). The recent rapid development of molecular sequencing technology has provided an effective way to combine the diversity of global species of symbiotic microbiota and the gut microbiotas of soil invertebrates. Previous studies reported that the gut microbiotas of soil invertebrates were mainly composed of the phyla Proteobacteria, Firmicutes, Actinobacteria, and Bacteroidetes [32], which are anaerobic and facultative anaerobic bacteria due to the special anaerobic environment of the gut.

**Fig. 4:**
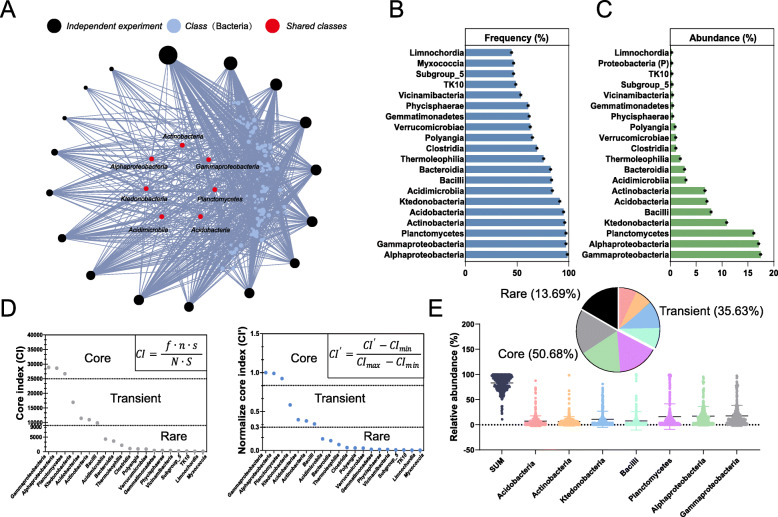
Defining the core microbiomes of the guts of soil invertebtrates based on a metadata analysis. Network analysis showing the unique and shared classes in the 17 independent experiments. The node size indicates the number of connections, the node color indicates the following: black nodes, different independent experiments; blue nodes indicate bacterial classes not shared between all experiments; red nodes indicate bacterial classes shared by all independent experiments (**A**). The frequencies (**B**) and average abundances (**C**) of the 20 most abundant classes in all samples, respectively. Core index (CI) for core, transient, and rare bacteria (at the class level) in the guts of the soil invertebrates (f, class frequency; n, number of independent experiments with class data; s, sequence length of the class; S, total sequence length from all samples) and NorCI, the normalized core index using all classes detected to normalize CI (**D**). Relative abundances of the core (50.68%), transient (35.63%), and rare (13.69%) classes in all samples (**E**). All data from the independent studies were merged into a single data set for all analyses

We unified the determination of the core gut microbiotas of soil invertebrates through collection of data from 33 independent experiments by searching the Web of Science Core Collection and Science Direct, 20 of which were publicly available and contained incomplete 16S rRNA gene sequences (*Data set* S1). We then reanalyzed these sequences and homogenized them to 2000 reads for each sample. The merged OTU table contained 415 gut samples from soil invertebrates in 17 independent experiments: nematodes, springtails, earthworms, mites, and ants (*Data set S*1). The gut microbiotas of soil invertebrates are contributed by host factors such as diet [33], habits [34], region [6], and soil type [35], so refining the core gut microbiotas of soil invertebrates at a higher level of classification, the class level, was necessary. First, the results of the taxa-detection rate for each experiment identified seven shared classes: Actinobacteria, Alphaproteobacteria, Ktedonobacteria, Acidimicrobiia, Acidobacteriae, Planctomycetes, and Gammaproteobacteria (Fig. 4A). We then analyzed the frequency and relative abundance of each class across all samples (Fig. 4B, C). The two indicators, frequency and relative abundance, had different rank orders. A core index (CI) was established for calculating the symbiotic potential of core microbiotas from all taxa based on a metadata analysis using Eq. 2 (*Supplementary Information*), and we used the normalized core index (NorCI) to determine the core gut microbiota of the soil invertebrates (Eq. 3, *Supplementary Information*) (Fig. 4D). According to the previous classification method, based on the NorCI thresholds of 0.83 and 0.3 (*Supplementary Information*) and the frequency of all samples, we further divided the bacterial classes into core (Gammaproteobacteria, Alphaproteobacteria, and Planctomycetes; 50.68%), transient (Bacilli, Ktedonobacteria, Actinobacteria, and Acidobacteria; 35.63%), and rare (lower-CI taxa; 13.69) gut microbiota (Fig. 4E). Interestingly, CI and NorCI for the invertebrates and the surrounding soil (Figure S10A) showed that the core taxa in soil were very similar to the transient taxa in the invertebrate gut. A PCoA based on the OTU data using Bray-Curtis distances, however, identified a pronounced separation between the soil invertebrates and the bacterial communities of the surrounding soil (Figure S10B). Surprisingly, CI was also highest for Gammaproteobacteria in the surrounding soil, but the PCoA based on Gammaproteobacteria OTU data demonstrated that the gut Gammaproteobacterial community differed from the community in the surrounding soil. This finding may have been due to the special anaerobic environment of the gut (Figure S10C).

### Screening the most important gut bacterial taxa that respond to pollutant pressure using a machine-learning method and associated community function

We screened data for a total of 415 gut samples (193 controls and 222 pollution groups) from 17 independent experiments. The samples were from earthworms, *Enchytraeus*, collembolas, ants, and mites exposed to 15 kinds of soil pollutants (e.g., the fungicide azoxystrobin; the insecticide cypermethrin; the herbicide glyphosate; the antibiotics tetracycline, sulfamethoxazole, and oxytetracycline; the antibiotic substitute *Macleaya cordata* extract; the heavy metals arsenic, silver nitrate, silver nanoparticles, and nano-copper oxide; and the emerging pollutants micro-, nano-, and tire-tread plastics), manure, and lime.

**Fig. 5:**
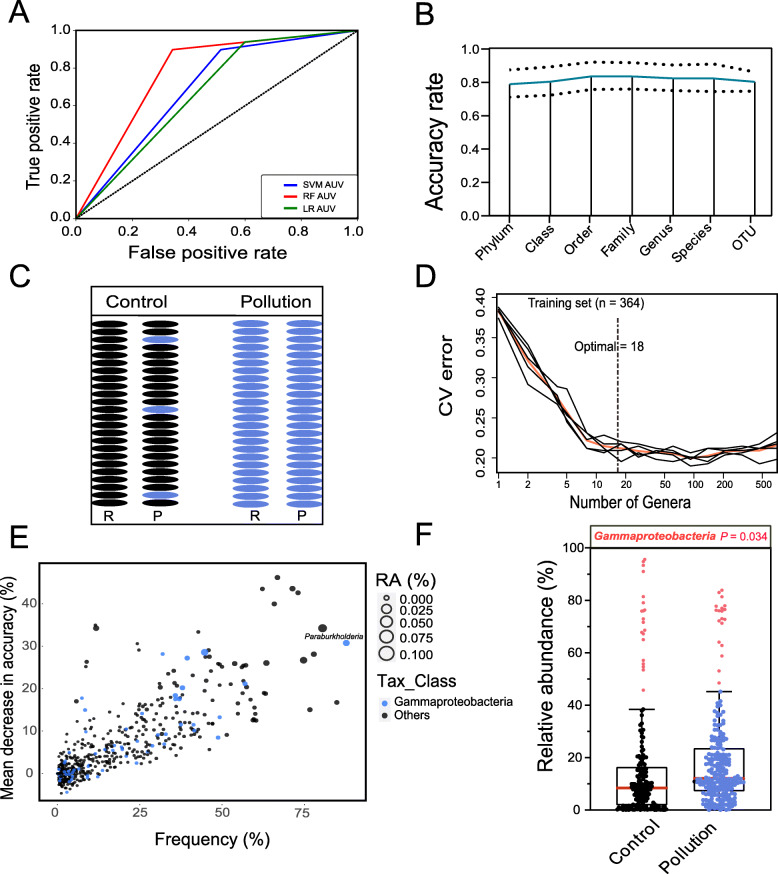
Screening of gut microbiota that responded to pollutant pressure using a machine-learning method. The machine-learning modules of the random forest (RF), support-vector machine (SVM), and logistic regression (LR) analyses were built using the OTU data for the bacterial community (**A**). The associated AUC and ROC curves indicated that the RF model was the most accurate model. The RF module constructed using all samples at the phylum, class, order, family, genus, and OTU levels. Dashed lines indicates the 95% confidence interval (**B**). Prediction using the test data in the RF module. Black indicates the control, and blue indicates the treated groups in the comparison between raw (O) and predictive (P) information (**C**). Each test used a tenfold cross-validation method to verify the accuracy of the model predictions, and the 18 most abundant bacterial genera were identified by applying the RF classification of the relative abundances of the control and treated samples (**D**). RF classification of the relative abundance of the control and treated samples as based on the data for all genera for calculating their mean decreases in accurracy, combined with the frequency of each genus for screening the most important indicator genus, *Paraburkholderia*, belonging to Gammaproteobacteria (**E**). The associated relative abundance of Gammaproteobacteria in the control and the treated groups (**F**). *P* values were determined using two-tailed Welch’s *t* tests

**Fig. 6:**
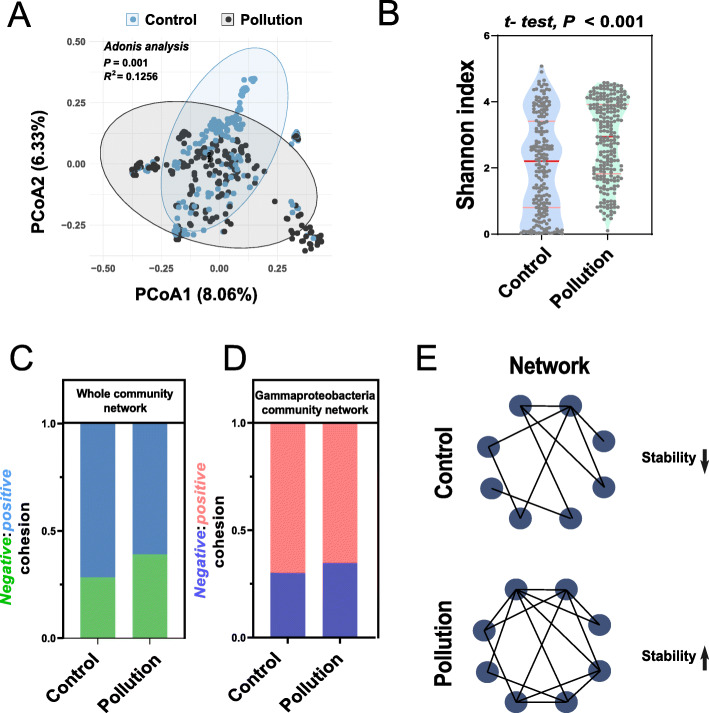
Effects for soil pollutant in stability of microbial networks of soil invertebrate gut. Principal coordinate analysis (PCoA) of the 17 independent experiments with 415 gut samples (193 control and 222 treated), including 17 kinds of soil pollutants (*Data set S**1*) using Bray-Curtis dissimilarity performed on OTU data from the control and treated microbial communities (**A**). *R*^2^ and *P* values were determined using Adonis analysis. Shannon index for the gut mirobial communities in the control and treated groups (**B**). *P* values were determined using two-tailed Welch’s *t* tests. *Negative*:*positive* cohesion (calculated using the formula in (28)) in the whole (**C**) and Gammaproteobacteria-associated communities (**D**) networks with and without soil pollution, indicating that the treated groups formed a more stable community network than the control as shown in the schematic diagram (**E**)

To determine whether gut bacterial taxa could be used as biomarkers for responses to soil pollutants, we built three machine-learning models, random forest (RF), support-vector machines (SVM), and logistic regression (LR), in which the accuracy rate (area under the curve) indicated that RF was the best model for predicting the classification of samples (Fig. 5A). We therefore calculated the accuracy of the classification based on the bacterial data at the levels of phylum, class, order, family, genus, species, and OTUs and selected the most accurate classification of the genus data to establish the RF model (Fig. 5B). The predication of this model based on the test data set was 100% accurate for the pollution samples and 84.2% accurate for the control samples (Fig. 5C). We next conducted a tenfold cross-validation with five repeats to evaluate the importance of genera as potential indicators (Fig. 5D), which combined with the frequency data for each genus, and confirmed the bacterial biomarker taxa (Fig. 5E) belonging to Gammaproteobacteria and their enrichment in contaminated guts (Fig. 5F). Meanwhile, taking the heterogeneity of different experimental pollutants, we used a meta-analysis and a sensitivity analysis based on a random model and found an upward trend of Gammaproteobacteria in the contaminated group (Figure S11). This finding supported the hypothesis that Gammaproteobacteria, by accumulating within the gut, could act as indicators of soil disturbance by pollution. A PCoA based on the OTU data from the rarefied sequencing reads for each gut sample using the Bray-Curtis distances presented significantly separated clusters between control and pollutant treatments (Fig. 6A: PERMANOVA for treatments: *R*^2^ = 0.1256, *P* < 0.001, Adonis analysis). Bacterial diversity (Shannon index) was higher in the pollutant treatments (Fig. 6B: *P* < 0.001, *df* = 415, two-tailed Welch’s *t* test), indicating that pollutant residues in the soil interfered with the structure and diversity of the gut microbial community. The topological properties analysis exhibited an insignificant change between control and treatment group (Figure S12). The *negative*:*positive* cohesion of each bacterial community and the Gammaproteobacteria-correlated community network in the control and pollutant treatments were calculated using the equation described by Hernandez et al. [27], to determine the function of the Gammaproteobacteria community in the gut. The higher *negative*:*positive* cohesion in the bacterial communities of the contaminated guts demonstrated that soil pollutants stimulated the gut to develop a more stable community network (Fig. 6C, E), especially the Gammaproteobacteria, which greatly promoted the stability of the gut bacterial community (Fig. 6D).

## Discussion

The rapid development of next-generation sequencing technology and bioinformatics has enabled large-scale, cross-influence analyses of complexity, which has greatly enlarged our understanding of the interaction between microbial communities and their ecological niches [36–38]. Interestingly, microbiotas directly and closely associated with personal, public, and planetary health [39] have gradually become indicators for predicting human and ecological health [17, 18, 37, 38, 40]. Our previous studies have found that the gut microbiota of soil invertebrates was more sensitive than soil microbiotas to soil pollution [14, 15, 34]. Identifying common potential indicator taxa in invertebrate guts may therefore be beneficial to the assessment of soil ecological risk. We used a microcosm experiment for prediction, and used metadata and machine learning for verification, to identify indicator taxa from the guts of soil invertebrates in response to environmental concentrations of soil pollution and their relationships with the associated gut microbiome and resistome and with characteristics of host physiology and biochemistry.

We exposed the model soil invertebrate *F. candida* to common soil pollutants (a fungicide (AZ) and an antibiotic (OTC)) in our microcosm experiment, separately targeting fungal and bacterial communities. The survival and fecundity of *F. candida* were not significantly affected, but the significant increase in ROS confirmed that the host was negatively affected by these pollutants (Fig. 1D). The enzymatic activity of CYP450 was also higher in the pollutant treatments, indicating a biochemical stress response and detoxification mechanism by *F. candida* [15]. This result indicated that soil pollutants at environmentally relevant residual concentrations could not be indicated via ordinary physiological phenomena. The HAA index also indicated that the effect of AZ and OTC was higher when combined, than individually (Fig. 1C), unlike the effect on the *F. candida* gut microbiota. A specific taxon rather than the entire bacterial community was thus likely an indicator of the response to soil pollution.

We could not differentiate the diversity and structure of the soil bacterial community between the control and pollutant treatments. The structure of the fungal community was significantly affected by soil pollution, but the fungal OTUs were not shared among the pollutant treatments. Interestingly, the gut bacterial community had a stress response to the pollutants similar to the host physiological response and also shared significantly different OTUs in each treatment group, suggesting that the gut and soil bacteria possessed common response characteristics to soil pollution, indicating that the gut bacteria may not have been disturbed by specific targets of the soil pollutants. The diversity of colonization by the fungal community in the gut of *F. candida* was strongly affected by dietary structure, but the potential for colonization was much lower for the fungi than the bacteria.

The gut bacteria of soil invertebrates may thus be suitable potential indicators of soil pollution. We classified the OTUs shared at the phylum and class levels as Proteobacteria and Gammaproteobacteria (Fig. 2E), which were significantly enriched in the *F. candida* gut in the pollutant treatments. Interestingly, the change in relative abundance of Gammaproteobacteria was consistent with the HAA index after exposure to OTC, AZ, and AO, supporting Gammaproteobacteria as a potential indicator of the response to soil pollution. We constructed the co-occurrence network, including both bacteria and fungi, to test the contributions of Gammaproteobacteria to the entire gut microbiota and found that Gammaproteobacteria correlated with most other bacteria and fungi to maintain the interoperability and intercommunications throughout the microbial community (Fig. 2G).

*F. candida* is sensitive to soil pollutants, which will inevitably affect the level of molecular functions. We used a WGCNA analysis of the transcriptomic data to identify the functional characteristics of the host under the pressure of soil pollution for determining the MEbrown co-expressed gene module, significantly correlated with the control phenotypes, which differed from the pollution phenotypes (Figure S8). Interestingly, the expression of genes significantly correlated with Gammaproteobacteria was downregulated in the pollutant treatments. These genes were assigned to immunological, digestive, metabolic, and other functional pathways essential to the host. We used SEM to identify the relationships between the host function and Gammaproteobacteria abundance, which again demonstrated that Gammaproteobacteria were negatively linked to host function (transcriptome data) by affecting the diversity of the gut bacterial community (Fig. 3G, H). The relative abundance of Gammaproteobacteria was significantly and directly correlated with the levels of oxidative stress and locomotive ability of the host. These results strongly supported Gammaproteobacteria as a potential indicator taxon in response to soil pollution, although limited to the environmental concentrations in this study.

Proteobacteria have been considered as gut microbiota associated with dysbiosis in humans, blooms of which have been correlated with obesity, diabetes, and some immunological disorders [30]. Proteobacteria are facultative anaerobic bacteria, whose unique oxygen consumption affects the gut environment, making it conducive to colonization by many types of anaerobic bacteria and destroys the stability of the original gut microbial community. The phylum Proteobacteria is generally considered to be comprised of 116 validated bacterial families and has the largest phylogenetic composition, with highly diverse morphological and physiological functions for maintaining a competitive advantage in adapting to complex and diverse ecological niches (e.g., soil [36], plants [41], freshwater [42], seawater [43], and the atmosphere [44]). In particular, one group of Proteobacteria, the Gammaproteobacteria, often aggressively occupies the ecological niche of the symbiotic relationship between plants and insects [45, 46]. We therefore hypothesized that Gammaproteobacteria would occupy an important core position in the gut microbiotas of soil invertebrates. We analyzed 16S rRNA gene sequences from the guts of 17 soil invertebrates available in public databases but found no consensus among previous reports for defining the core microbiota. A current strict definition for an indicator species is that all samples must include a specific taxon (at the OTU level) [47], but this definition is not suitable for many distantly related host species [10, 48]. Various independent studies have included different test species, environments, and methods, especially those based on metadata analyses, which may be less appropriate, because the number of shared OTUs may be much lower than at other levels of classification.

We thus took into account both the percent occurrence in samples and the relative abundance of each taxon [16] and used class as the core taxonomic level. We also constructed a shared network based on each independent experiment, identifying the classes shared among all studies (Fig. 3*A*), to avoid interference of the variation of different independent experiments. Based on the above factors, we developed an equation using CI to represent the roles of each taxon and identified the core taxa based on the metadata analysis (Eq. 2, *Supplementary Information*). Gammaproteobacteria had the highest CI, supporting their role as an important core taxon in the guts of soil invertebrates. Other core gut taxa (Alphaproteobacteria and Planctomycetes) with a total relative abundance 50.68% were identified using 0.83 as the CI’ threshold. A CI’ threshold of 0.3 was used to identify the transient taxa (35.63%): Bacilli, Ktedonobacteria, Actinobacteria, and Acidobacteria (Fig. 2E). Previous studies also defined the core taxa of common soil invertebrates. They included *Rickettsia* and *Pseudomonas* in *Orchesella cincta* and *Folsomia candida*, *Enterobacteriaceae*, *Pseudomonadaceae*, and *Sphingomonadaceae* in *Caenorhabditis elegans*, all of which are classified to the Gamma- and Alpha-proteobacteria [49], and these taxa also strictly conformed to our definition. The definition of the core taxa of the same genetic relationship was conserved at the family level, but the cross-species core taxa definition in this study can only be conserved at the class level. The metadata analysis also indicated that gut core taxa have similar core taxa classification characteristics, but the specific species composition and structure are significantly different, indicating that the assembly of the gut microbiotas of soil invertebrates depended on the host and deterministic processes, perhaps associated with the structure and function of the gut. These results provide a basis for future research on the microbial diversity of soil ecosystems.

We divided a total of 415 samples, from 17 independent experiments, into two parts, control and pollutant treatments. The diversity and structure of the bacterial community differed significantly between the controls and pollutant treatments, and the bacterial communities of the contaminated guts were more stable (Fig. 6), indicating that higher bacterial-community diversity can maintain the stability of community function. Previous studies have reported that soil invertebrates will only accumulate a small amount of pollutants in their guts [14], leaving the gut community under sub-stressed conditions, which could reduce competition between taxa and increase bacterial diversity. The Intermediate Disturbance Hypothesis [50] states that appropriate interference prevents competitive exclusion in communities [51], indicating that coerced systems are more stable than uncoerced systems. The gut bacterial communities in our study had a positive defensive strategy under pollution stress, and that Gammaproteobacteria played a key role in maintaining the stability of the communities. Due to the complex anaerobic environment of the gut and its high species heterogeneity, finding an effective method for determining the action of Gammaproteobacteria on the host, such as the commonly used verification methods, isolation of gut microbiota, and artificial construction of functional microbiota [52, 53], is difficult. The construction of sterile gut environments in our study has especially identified large obstacles to the verification of the specific identity of Gammaproteobacteria. The combination of metadata analysis and machine learning is a good alternative to laboratory-validated methods by building models using large-scale computational analysis. The RF model we used in this study has been widely used in the prediction of different communities, such as distinguishing between the gut bacterial communities of mice that feed on normal diets and those with high salt content [54], distinguishing between soil fungal and bacterial communities with and without fusarium wilt [55] and distinguishing between the root microbial communities of indica and japonica rice [18]. Interestingly, we also found that the healthy and contaminated guts were accurately distinguished using an RF model, which can be built at any taxonomic level with similar average accuracy rates, so we selected the RF model at the genus level with the lowest estimated rate of out-of-bag (OOB) errors (17.58%), indicating that Gammaproteobacteria was the core taxon that may respond to soil pollutants and was enriched in the contaminated guts of the soil invertebrates (Fig. 5E, F).

Most interestingly, previous meta-analyses of gut microbiota showed that Gammaproteobacteria (*Escherichia coli*) may be the main ARBs in the human gut [56]. We detected that Gammaproteobacteria was the only bacterial class that was significantly and strongly correlated with the relative abundance of ARGs (Figure S13), indicating that Gammaproteobacteria may affect the gut resistome of *F. candida*. The soil invertebrate gut is an important reservoir for ARGs, transferring them across the soil food web, thus allowing its use as one of the indicators for risk assessment of soil ecology and health [57]. Therefore, Gammaproteobacteria not only respond to the environmental concentration but can also reflect the dynamic changes among ARGs, which may allow evaluation of soil ecological health risks from two perspectives. It is therefore a reasonable and flexible strategy to construct Gammaproteobacteria general-purpose primers, to provide an indication of soil ecological and health risks.

## Conclusion

We combined our microcosm experiment, metadata analysis, and a machine-learning method to identify the core taxa in the guts of soil invertebrates and provided a quantitative method for identifying core taxa in microbial communities based on metadata analysis that is suitable for different habitats and species. Interestingly, we also determined that Gammaproteobacteria were a potential indicator taxon in the guts of the soil invertebrates that responded to environmental concentrations of soil pollutants, thus providing an effective theoretical basis for subsequent assessments of soil ecological risk. Additionally, the results from the physiological and biochemical analyses of the host, and the microbial-community functions and antibiotic resistance of Gammaproteobacteria, also provide new insights for evaluating global soil ecological health.

## Supplementary Information

**Additional files 1: Extended Methods.** Test soil, species, and pollutants; Laboratory experimental design; DNA extraction, DNA amplification, library preparation, sequencing and bioinformatic analysis; RNA isolation, transcript sequencing, library preparation and bioinformatic analysis; Data collection and description and processing of the 16S rRNA metadata; Construction and validation Prediction model; Equation for the high area activity (HAA) index and core index (CI).

**Additional files 2: Figure S1.** Graphic representation of the experiment and analysis method. **Figure S2.** Number of adults in the control and all treatments. **Figure S3.** Alpha diversity (Shannon and Chao indexes) and beta diversity of the bacterial and fungal communities in the soil surrounding *Folsomia candida*. **Figure S4.** Number of significantly different fungal OTUs in all treatments compared to the control group. **Figure S5.** Richness (Chao index) of the bacterial and fungal communities in the gut of *Folsomia candida*. **Figure S6.** Alpha diversity (Shannon and Chao indexes) and beta diversity of the fungal community in the gut of *Folsomia candida*. **Figure S7.** Stability of the networks of interaction between bacteria and fungi in all groups. **Figure S8.** The degrees and closeness centrality of bacterial classes (relative abundance) in bacteria-bacteria and bacteria-fungi co-occurrence networks from all laboratory samples. **Figure S9.** WGCNA analysis of *Folsomia candida*. **Figure S10.** Core index (CI) and normalized CI (NorCI) of the bacterial communities in the soil surrounding the soil invertebrates across eight independent experiments. **Figure S11.** Meta-analysis and sensitivity analysis of Gammaproteobacteria relative abundance in soil invertebrate guts. **Figure S12.** The topological properties (degrees, closeness centrality, and betweenness centrality) of bacterial classes in the co-occurrence networks of control and pollution group from all metadata samples. **Figure S13.** The identification of antibiotic resistance bacteria (ARB) in soil invertebrate gut. **Figure S14.** Annotation of all mapped genes using the GO, KEGG, COG, NR, Swiss-Prot, and Pfam databases.

**Additional files 3: Data set S1.** Uploading sequence data ID. **Data set S2.** Antibiotic resistance gene primers.

## Data Availability

All meta-analysis sequencing data used here were either obtained from publicly accessible databases or directly from the authors of the studies, and the Uploading sequence data IDs are shown in *Data set S**1*. The laboratory experiment sequencing data has been deposited in the National Center for Biotechnology Information Sequence Read Archive (SRA) database (accession number: PRJNA743602).

## Abbreviations

ARG: Antibiotic resistance gene
CI: Core index
OECD: Organization for Economic Cooperation and Development
ITS: Internal transcribed spacer
RF: Random forest
LR: Logistic regression
SVM: Support-vector machine
ROC: Receiver operating characteristic curve
PCoA: Principal coordinate analysis
HAA: Index of high area activity
ROS: Reactive oxygen species
CYP450: Cytochrome P450
OTUs: Operational taxonomic units
WGCNA: Weighted gene co-expression network
KEGG: Kyoto Encyclopedia of Genes and Genomes
PERMANOVA: Permutational analysis of variance
RIN: RNA integrity number
TPM: Transcripts per million
SEM: Structural equation modeling
GBIF: Global biodiversity information facility
NorCI: Normalized core index
OOB: Out-of-bag
