# Supplementary material for: Gammaproteobacteria, a core taxon in the guts of soil fauna, are potential responders to environmental concentrations of soil pollutants
Source: Microbiome. 2021 Sep 30;9:196. doi: 10.1186/s40168-021-01150-6 (PMC8485531; doi:10.1186/s40168-021-01150-6)
Supplement: Supplementary file 2 — Additional files 1: Extended Methods. Test soil, species, and pollutants; Laboratory experimental design; DNA extraction, DNA amplification, library preparation, sequencing and bioinformatic analysis; RNA isolation, transcript sequencing, library preparation and bioinformatic analysis; Data collection and description and processing of the 16S rRNA metadata; Construction and validation Prediction model; Equation for the high area activity (HAA) index and core index (CI). [file 40168_2021_1150_MOESM2_ESM.docx]

**Gammaproteobacteria, a core taxon in the guts of soil fauna, are responders to environmental concentrations of soil pollutants**

Qi Zhang^1^, Zhenyan Zhang^1^, Tao Lu^1^, Yitian Yu^1^, Josep Penuelas^2,3^, Yong-Guan Zhu^4,5^, Haifeng Qian^1, ^[[1]](#footnote-1)^*^

1. College of Environment, Zhejiang University of Technology, Hangzhou 310032, P. R. of China.
2. CSIC, Global Ecology Unit CREAF- CSIC-UAB, Bellaterra, Barcelona 08193, Catalonia, Spain.
3. CREAF, Campus Universitat Autònoma de Barcelona, Cerdanyola del Vallès, Barcelona 08193, Catalonia, Spain.
4. Key Laboratory of Urban Environment and Health, Institute of Urban Environment, Chinese Academy of Sciences, Xiamen 361021, P. R. of China.
5. State Key Lab of Urban and Regional Ecology, Research Center for Ecoenvironmental Sciences, Chinese Academy of Sciences, Beijing 100085, P. R. of China.

**Extended Methods**

**Test soil, species, and pollutants**

The test soil was a sandy loam (2.27, 16.47 and 81.25% of clay, silt, and sand, respectively) collected from the topsoil layer (0-20 cm) in a vegetable field (29°49′N, 121°20′E; Zhejiang, China), and its properties were as follows: pH (CaCl_2_), 5.16; water-holding capacity, 46.8%; and total N content, 3.8 g/kg. The soil was air-dried in the shade, and root debris, stones, and other allogenic materials were removed before sieving through a 0.2-mm mesh plastic soil sieve.

The test species, *Folsomia candida* (“Berlin strain”, originally obtained from Aarhus University, Denmark), was reared in our laboratory for two years following Organization for Economic Cooperation and Development (OECD) guideline 232 [1] and was placed a suitable breeding environment, maintained in Petri dishes containing a mixture of charcoal and plaster of Paris (1:8 w/w). These Petri dishes were kept in a thermostatic box (Safe Co., Ningbo, China) at 75% relative humidity and a temperature of 20 ± 2 °C with a 16:8 h light: dark photoperiod (800 Lux). Ultrapure water was added once a week to ensure substrate was moist and the *F. candida* were fed twice a week with dry yeast powder.

Azoxystrobin (AZ, C_22_H_17_N_3_O_5_, CAS 131860-33-8, white powder, 95% pure) and oxytetracycline (OTC, C_22_H_24_N_2_O_9_, CAS 79-57-2, crystalline powder, 97% pure) were purchased from Aladdin (Shanghai, China). AZ and OTC were diluted in methanol: ultrapure water (1:5 Volume/volume), and 0.1 g/L stock solutions were prepared. For both pre-exposure and formal experiment, we selected the environmental concentration of OTC and AZ as test concentration [2-8]

**Laboratory experimental design.**

To obtain *F. candida* all of the same age, we transferred 60-70 active adults to a new substrate to lay eggs for 2 days, then removed these adults. As the juveniles hatched from the eggs, they were immediately transferred to a new substrate for culture. Before the exposure experiment, 7-9-day-old collembolans were transferred to the test soil to adapt the test collembolans to the new culture environment.

When the mortality of these collembolans in the new soil culture environment was less than 1% during the pre-incubation process, we used these collembolans for subsequent exposure experiments. To simulate the natural field environment, we did not feed in the pre-incubation and exposure experiments and added distilled water twice weekly to maintain soil moisture.

We firstly conducted a pre-exposure experiment to measure the reproduction rate and mortality, for selecting the most suitable concentration for the formal experiments. Soil microcosms were established for exposing *F. candida* to the pollutants: control, AZ (0.3, 3, and 30 mg AZ/kg dry soil), OTC (10 mg/kg dry soil), and AO (combined 0.3 mg AZ/kg dry soil and 10 mg OTC/kg dry soil, 3 mg AZ/kg dry soil and 10 mg OTC/kg dry soil, and 30 mg AZ/kg dry soil and 10 mg OTC/kg dry soil). Twenty 14-16-day-old pre-incubated collembolans were introduced into these experimental microcosms. Each treatment had four replicates and were conducted in sterile glass beakers (inner diameter 5.3 cm, 6.5 cm high) containing 30 g moist soil, and incubated at 18 °C with a diurnal light cycle (16:8 h light:dark). We added sterile water twice a week and recorded the numbers of adults in all microcosms after 28 d of exposure using the imageJ 1.52.

The formal laboratory experiments were divided into two parts, one for determining the death, reproduction, locomotion, ROS concentration, and CYP450 enzymatic activity of *F. candida*, and the other for transcriptomic measurements (gene expression), the gut microbiota (bacterial and fungal communities), and gut resistome of *F. candida*. Before starting the experiments, we added ultrapure water to restore the microorganisms in the soil for a week at 20 ± 2 °C, and the moisture content was maintained at 60% of the maximum (60.23%), as detailed in Organization for Economic Cooperation and Development (OECD) guideline 232. In one part of the experiment, soil microcosms were established for exposing *F. candida* to the pollutants: control, AZ (0.3 mg AZ/kg dry soil), OTC (10 mg/kg dry soil) and AO (combined 0.3 mg AZ/kg dry soil and 10 mg OTC/kg dry soil). Twenty 14-16-day-old pre-incubated collembolans were introduced into these experimental microcosms, each microcosms had four replicates and were conducted in sterile glass beakers (inner diameter 5.3 cm, 6.5 cm high) containing 30 g moist soil at 18 °C with a diurnal light cycle (16:8 h light: dark). We added sterile water twice a week and recorded the numbers of adults and larvae in all microcosms after 28 d of exposure using the imageJ 1.52, and 13 adults were collected for the locomotory test. Two adults were then isolated for determining ROS content and CYP450 enzymatic activity respectively, following the manufacturer’s instructions for the nematode ELISA kit based on the double-antibody sandwich method (Jiangsu Enzyme Industrial Co., Ltd, Yancheng, China).

In the other experimental setup, sixty 14-16-day-old pre-incubated collembolans were added in control, AZ, OTC, and AO experimental microcosms with eight replicates. These experimental microcosms were conducted in the same sized beaker with 65 g moist soil. After exposure for 28 days, all collembolans were collected for gut microbiome, resistome, and eukaryotic transcriptome analysis. Fifty adults per replicate were washed three times with ultrapure water and dissected under a stereo microscope using very precise tweezers to obtain the collembolan gut samples [20].

The gut samples were transferred to 2-mL round-bottomed centrifuge tubes containing 20 μL of proteinase K and 180 μL of a lysis buffer solution for the extraction of DNA [9]. A total of 2 g of soil per sample, without collembolans, was collected for analyzing the soil microbial community. The other four replicates of each group provided fifty adults for RNA extraction. These collected collembolans were firstly washed three times with ultrapure water and then immediately frozen in liquid nitrogen for RNA extraction.

**DNA extraction, DNA amplification, library preparation, sequencing and bioinformatic analysis**

High-quality DNA from the *F. candida* guts (50 individuals) and soil samples (0.5 g) was isolated using a DNeasy^®^ Blood & Tissue Kit (QIAGEN, Dusseldorf, Germany) and a FastDNA^®^ Spin Kit for Soil (Mpbio InC, Santa Ana, USA), respectively, following the manufacturers’ instructions. The concentration and quality of the isolated DNA were then checked by agarose gel electrophoresis and ultraviolet absorbance (ND1000, Nanodrop, Thermo Fisher Scientific Inc.). The DNA was stored at -20 °C until further testing.

The V4 hypervariable region of the 16S rRNA gene was amplified using universal primers (forward primer 515F 5′-GTGCCAGCMGCCGCGG-3′ and reverse primer 806R 5′- GGACTACNVGGGTWTCTAA-3′) [9], and region 1 of the internal transcribed spacer (ITS) gene was amplified using the forward primer ITS1F (5′-CTTGGTCATTTAGAGGAAGTAA-3′) and the reverse primer ITS2 (5′-GCTGCGTTCTTCATCGATGC-3′) [10]. Although there still are some limitations in obtaining invariable taxa information when using these regions and primers, good reproducibility of the microbial community is only possible through the use of optimal and consistent amplification regions. The 50-μL reactions (25 μL of TaKaRa ExTaq DNA polymerase, 1 μL of DNA (range 10-15 μg/mL), 1 μL of universal forward, 1 μL reverse primer, and 22 μL of PCR-grade water) were amplified using the reaction conditions previously described [9]. The PCR products were then purified, pooled, and sequenced using the Illumina MiseqPE300 platform (Meiji, Shanghai, China).

We used Quantitative Insights Into Microbial Ecology (QIIME) to analyze the high-throughput sequencing data following the online instructions. Paired reads were spliced and merged into a single sequence using FLASH 1.2.11[11] based on the overlaps among the paired-end reads, and the quality of the reads and the sequences were controlled for quality and filtered (Fastp 0.19.6) [12]. Nonrepetitive sequences were then extracted from the optimized sequences, and singletons were removed. The nonrepetitive sequences were clustered by OTU at 97% similarity, and a representative OTU sequence was obtained after removing chimeras using Uparse 7.1 [13]. To obtain species information corresponding to each OTU, we used the ribosomal database project (RDP) classifier Bayesian algorithm (version 2.11) [14] to perform a taxonomic analysis of the representative OTU sequences based on a 16S rRNA database (SILVA 132) [15] and a fungal ITS database (UNITE 8.0)[16]. The alpha- and beta-diversities were determined using the Shannon and Chao indices and a principal coordinate analysis (PCoA) based on unweighted UniFrac distances, respectively.

**RNA isolation, transcript sequencing, library preparation and bioinformatic analysis**

A total of 50 adults per replicate were immediately snap frozen in liquid nitrogen and stored at -80°C to ensure the RNA integrity. The RNA was isolated from each replicate pool using an RNA extraction kit (HiPure Universal RNA Midi Kit, Magen, Guangzhou, China). The concentration and purity of the RNA were then determined using a Nanodrop2000 spectrophotometer, and an agarose gel electrophoresis was used to check the integrity of the RNA. An Agilent 2100 Bioanalyzer system was used to determine the RNA integrity number (RIN) value to meet the criteria for library preparation: total RNA ≥1 μg, concentration ≥35 ng/μg, OD260/280 ≥1.8 and OD260/230 ≥1.0. We used Oligo (dT) magnetic beads to isolate mRNA from the total RNA. The enriched mRNA was then randomly broken into fragments of ~300 bp and reverse transcribed to produce stable double-stranded cDNA using ReverTra Ace qPCR RT Kit (TOYOBO, Osaka, Japan)), which was sequenced using the Illumina Novaseq 6000 platform (Meiji, Shanghai, China).

The raw sequencing data were filtered using SeqPrep (<https://github.com/jstjohn/SeqPrep>) and Sickle (https://github.com/najoshi/sickle) to obtain clean data and ensure the accuracy of subsequent analyses of biological information. These clean data (reads) were mapped using the *F. candida* genomic database in NCBI (GCF_002217175.1) using TopHat2 and HISAT2 (http://blog.biochen.com/archives/337), and the mapping value (total mapped) was >97%, indicating that the experimental samples were not contaminated and that the reference genome was complete. The mapped data were then annotated using the GO, KEGG, COG, NR, Swiss-Prot, and Pfam databases (Fig. S14). To subsequently identify differences in gene expression between the groups, we used RSEM to calculate the read counts of mapping data and converted to standardized TPM values.

**Data collection and description and processing of the 16S rRNA metadata.**

Based on a previous search principle [17], we searched the Web of Science Core Collection and Science Direct for the terms “gut microbiota of soil fauna or ‘species’ name” “gut microbial community of soil fauna or ‘species’ name” and “gut microbiota of soil animal or ‘species’ name”. A total of 33 studies were collected from these databases, but only 20 independent experiments were publicly available and incomplete for 16S rRNA gene sequences (*Data set S1*), including three 16S rRNA gene hypervariable regions, V4, V4-V5 and V3-V4, which were amplified using the respective primer pairs 515F/806R (forward primer: 5′-GTGCCAGCMGCCGCGGTAA-3′, reverse primer: 5′- GGACTACNVGGGTWTCTAA -3′), 515F/907R (forward primer: 5′-GTGCCAGCMGCCGCGGTAA-3′, reverse primer: 5′-CCGTCAATTCMTTTRAGTTT-3′) and 334F/806R (forward primer: 5’-CCTACGGGAGGCAGCAG-3’, reverse primer: 5’-GGACTACHVGGGTWTCTAAT-3’). We first combined the raw 16S rRNA paired-end sequences from each independent experiment, excised the primer sequences to complete the quality control and limited read errors to <1%. These filtered sequences were redundant, so the OTU table was generated by clustering to ensure 97% accuracy. We next used the RDP Gold database to remove the chimeras and create the final OTU table. These operations were performed using Vsearch 2.7.1. To merge all 16S rRNA sequences, we constructed a feature table and a feature sequence format to import into QIIME2 (version 2021.2). We then classified these merged reference sequences using a classifier trained on the SILVA v132 database of full-length 16S rRNA gene sequences. The classified sequences were filtered to remove mitochondrial and chloroplast sequences, retaining only sequences from the kingdom Bacteria. Finally, and a total of 415 samples from the 17 independent studies, including the 17 soil pollutants (e.g. the fungicide azoxystrobin, the insecticide cypermethrin, the herbicide glyphosate, the antibiotics tetracycline, sulfamethoxazole and oxytetracycline, the antibiotic substitute *Macleaya cordata* extract, the heavy metals arsenic, silver nitrate, silver nanoparticles and nano-copper oxide and emerging pollutants micro-, nano- and tire-tread plastics), five kinds of soil invertebrates (collembolans, enchytraeus, earthworms, mites, and ants) and three exposure methods (oral exposure, soil microcosm, and field experiment). All metadata were divided into “Control” and “Pollution” based on the sample information for each experiment (*Data set S1*).

**Effects of soil pollution on *F. candida* gut resistome.**

We used a total of 384 primer sets (*Data Set S2*) (including 320 ARGs, 57 mobile genetic elements (MGEs), and the 16S rRNA gene) to investigate the composition and abundance of ARGs in the collembolan gut with the SmartChip Real-time PCR system (Wafergen, USA). The PCR reaction mixture of each well was composed of collembolan DNA template, primers, sterile water and LightCycler 480 SYBR Green I Master mix. The HT qPCR reaction conditions were: 95 °C, 10 min and 40 cycles of 95 °C, 0.5 min and 60 °C, 0.5 min. SmartChip qPCR software was used to analyze the raw data and a threshold cycle (CT) of 31 was used to detect ARGs. Only when 3 technical replicates and 3 biological replicates were amplified at the same time, did we consider an ARG to have been detected. The relative abundance of ARGs were calculated using the equation below [18].

$$Copy number of gene={10}^{((31-CT\left( measurement \right))/(10/3)}$$

**Construction and validation Prediction model.**

Machine-learning algorithms were used to predict the heterogeneous microbial data for identifying taxa in the guts of the soil invertebrates that were most strongly associated with the stress of soil pollutants. We utilized three machine-learning algorithms, random forest (RF) [19], logistic regression (LR) [20], and support-vector machine (SVM) [21]. We first evaluated the performance of the classifier by five-fold cross-validation of the training set and then selected the smallest absolute contraction and selection operators in the five iterations. The metadata from the 17 independent experiments (415 samples) were divided into two sets: 90% for the training set and 10% for the test set. The training set was randomly divided into five equal portions. Eighty percent of the training set was selected to train the classifiers, which was used for the prediction or validation of the remaining 20%. The receiver operating characteristic curve (ROC) and the area under the curve indicated that the RF algorithm performed well on our merged data. We therefore used the RF algorithm to build the predictive model. Classification models based on each taxonomic level could distinguish between the bacterial communities in the guts of the soil invertebrates in the control and pollutant treatments using the randomForest package in R with default parameters (version 4.6-14). The results indicated that the average accuracy rate was similar across all taxa, so we selected the RF model at the genus level with the lowest estimated rate of out-of-bag (OOB) errors (17.58%) [22].

**Equation for the high area activity (HAA) index and core index (CI)**

The HAA index was used to characterize the locomotive ability of *F. candida* in the laboratory experiment:

$HAA=\frac{N_{A1-3}}{N_{A4-5}}$ (1)

where **HAA** is the high area activity index of *F. candida*, **N_A1-3_** is the sum of individual numbers in map areas A1, A2, and A3 and **N_A4-5_** is the sum of individual numbers in areas A4 and A5.

CI was established for calculating the symbiotic potential of core microbiota from all taxa based on the metadata analysis:

$CI=\frac{f*n*s}{N*S}$ (2)

where **CI** is the core index of each class in the 16S rRNA sequencing metadata, **f** is the frequency of each class in all samples, **n** is the number of independent experiments that included each class, **s** is the sequence length of each class in all samples, **N** is the total number of individual experiments and **S** is the total sequence length from all samples.

We used the normalized core index (NorCI) to determine the colonization potential of core microbiota in the guts of the soil invertebrates:

$NorCI=\frac{CI'-{CI}_{min}}{{CI}_{max}-{CI}_{min}}$ (3)

where **CI'** is the core index of each class CI in soil invertebrate gut, **CI_min_** and **CI_max_** are the minimal and maximal CI in this metadata for the gut microbiota of the soil invertebrates, respectively).

The use of the most logical selection threshold usually depends on the aim of a study [23], so we combined the microbiota data shared by each experiment and set the threshold of NorCI to 0.3 for the exposure methods, soil types, and species differences in each individual experiment.

**Statistical methods.**

The means ± standard errors (SEs) of each treatment were calculated. A two-tailed Welch’s *t*-test was used to identify significant differences between groups. A principal coordinate analysis (PCoA) based on unweighted UniFrac distances for the guts of the soil invertebrates and for the bacterial and fungal communities in the surrounding soil was performed using the Majorbio Cloud Platform ([www.majorbio.com](http://www.majorbio.com)), and the output was visualized using OriginPro 9.1. The Adonis function (9999 permutations) was used in a PERMANOVA to identify differences among the treatments using the vegan 2.4-3 package in R version 3.6.1. Function prediction analysis of fungi using FUNGuild was performed on Majorbio Cloud Platform ([www.majorbio.com](http://www.majorbio.com)).

Heatmaps were generated using TBtools (Toolbox Biologists v0.655), and histograms, line and box charts, and linear regressions were produced using GraphPad Prism 8.00. The weighted gene co-expression network analysis (WGCNA) analysis was performed using the Majorbio Cloud Platform ([www.majorbio.com](http://www.majorbio.com)). The co-occurrence network analysis of laboratory experiments, based on the relative abundance of all bacterial and fungal classes, using pairwise Spearman’s rank correlations (r) in the psych package in R (*r* > 0.6 or *r* < -0.6, *P* < 0.05), was performed using Gephi v0.9.2. The shared network based on the frequency of each bacterial class among all independent studies was produced using Gephi v0.9.2. Structural equation models (SEMs) were built to calculate the direct and indirect effects among the gut bacteria (Shannon index), treated groups, bacteriaShannon/fungiShannon (B/F) index, cytochrome P450 (enzymatic activity), the HAA index, Gammaproteobacteria (relative abundance), transcriptome (PC1 of the TPM value using Bray-Curtis distances). The significance of each path-coefficient was analyzed by calculating its critical ratio (*P* < 0.05). The goodness-of-fit index (GFI) and the Bentler comparative fit index (CFI) indicating the goodness-of-fit of the models to the original data. The SEM was produced using Amos Graphics v22 (IBM Corp., Armonk, NY, USA). Meta-analysis and sensitivity analysis were performed using the STATA statistical software package version 15.0 (Stata Corp, College Station, TX, USA). In addition, to characterize positive and negative co-occurrences separately, we used the cohesion among taxa to reveal the interactions, similarity, and differences between both positive and negative species interactions in the niches of microbial taxa, using the previous equation to calculate the positive and negative cohesion values [27].


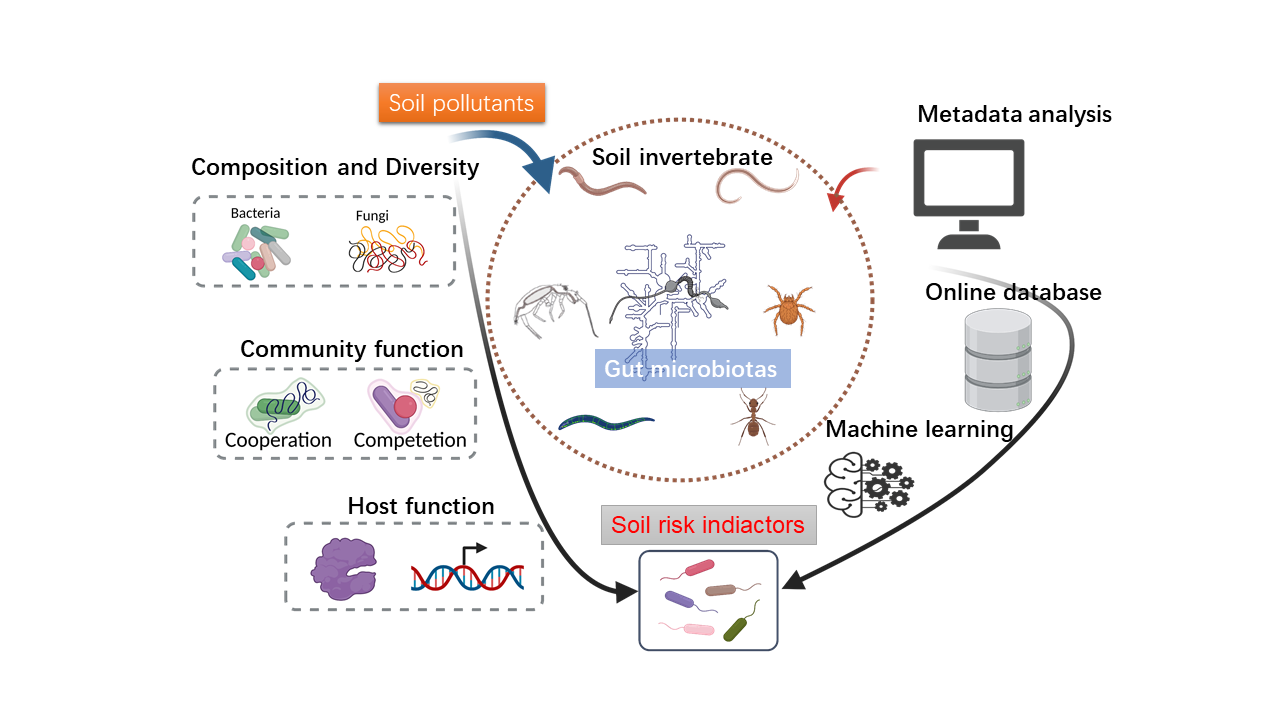


**Figure S1.** Graphic representation of the experiment and analysis method.


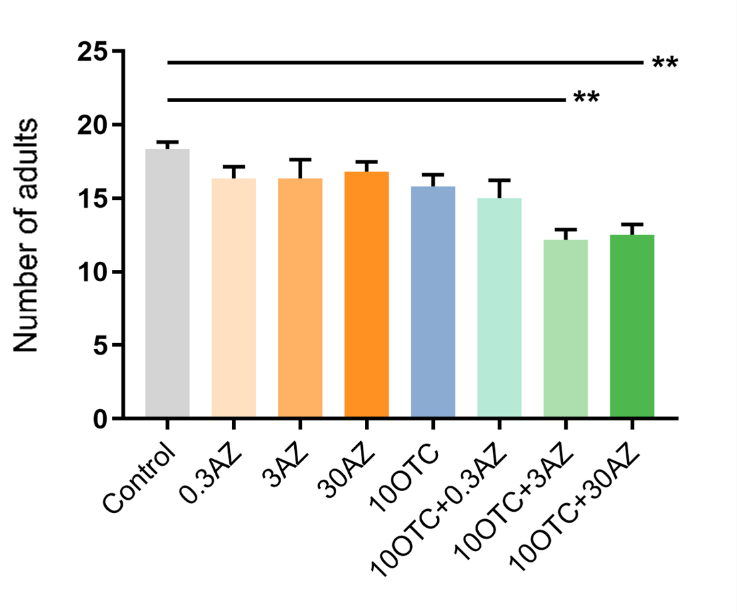


**Figure S2.** Number of adults in the control and the treatments with 0.3, 3, and 30 mg azoxystrobin (AZ)/kg dry soil (0.3AZ, 3AZ, and 30AZ), 10 mg oxytetracycline (OTC)/kg dry soil (10OTC), and 0.3, 3, and 30 mg AZ + 10 mg OTC/kg dry soil (10OTC+0.3AZ, 10OTC+3AZ, and 10OTC+30AZ). ** (*P* < 0.01) indicates significant differences between the control and the treatments (two-tailed Welch’s *t*-test).


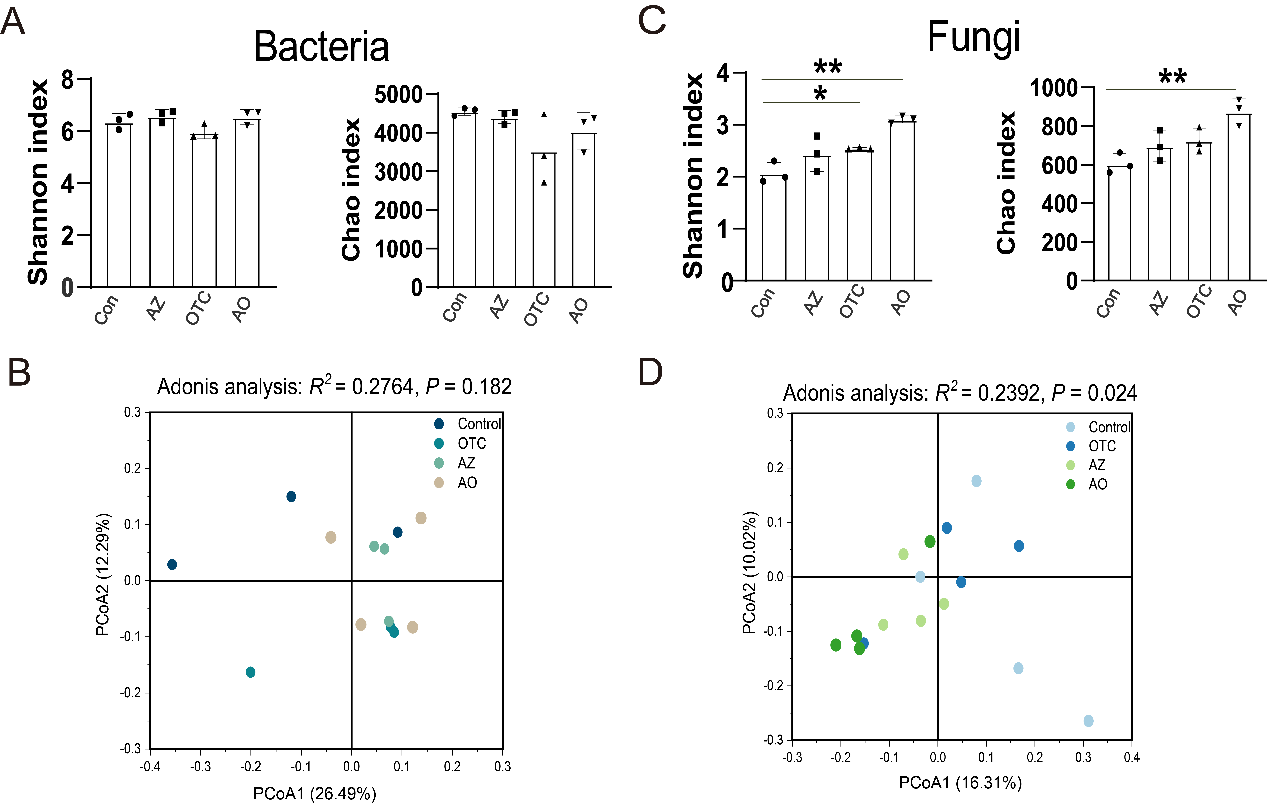


**Figure S3.** Alpha diversity (Shannon and Chao indexes) of the bacterial and fungal communities in the soil surrounding *Folsomia candida* after exposure to 0.3 mg Azoxystrobin (AZ)/kg dry soil, 10 mg oxytetracycline (OTC)/kg dry soil, and 0.3 mg AZ + 10 mg OTC/kg dry soil (AO) (**A**, **C**). A principal coordinate analysis (PCoA) and a multivariate PERMANOVA using unweighted UniFrac distances based on the relative abundances of soil bacterial and fungal OTUs were performed to account for the differences in the patterns of distribution of the OTUs in the surrounding soil (**B**, **D**). “*” and “**” (*P* < 0.05 and *P* < 0.01, respectively) indicate significant differences between the control and the treatments (two-tailed Welch’s *t*-test).


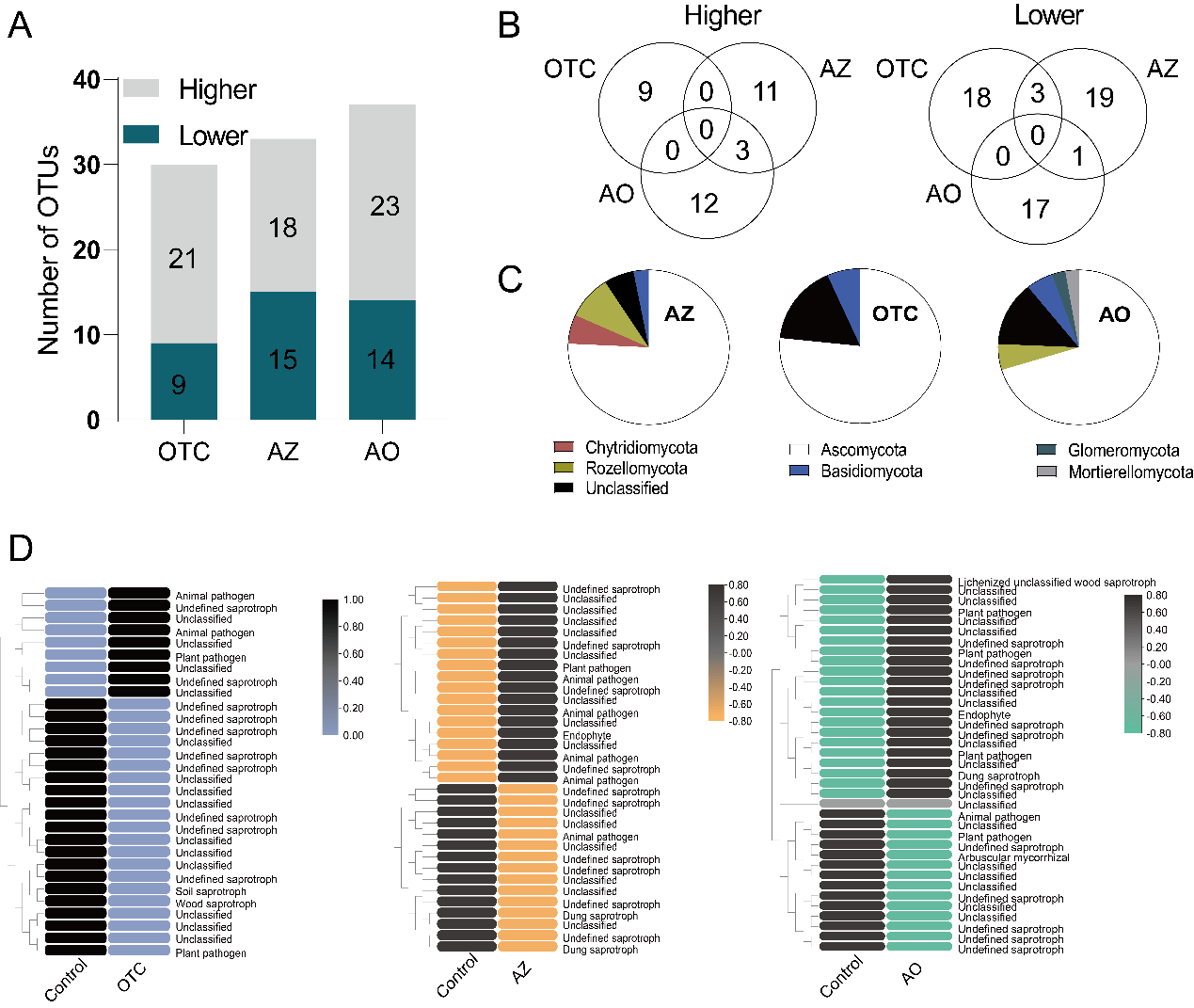


**Figure S4.** Number of significantly different fungal OTUs in the OTC, AZ, and AO groups compared to the control (MetagenomeSeq analysis) (**A**). The gray and green columns indicate OTUs with lower and higher relative abundances, respectively. The numbers and taxa of shared significantly different OTUs among the OTC, AZ, and AO groups (**B**, **C**). Heatmaps of the functional classification of the significantly different fungal OTUs in the OTC, AZ, and AO groups (**D**).


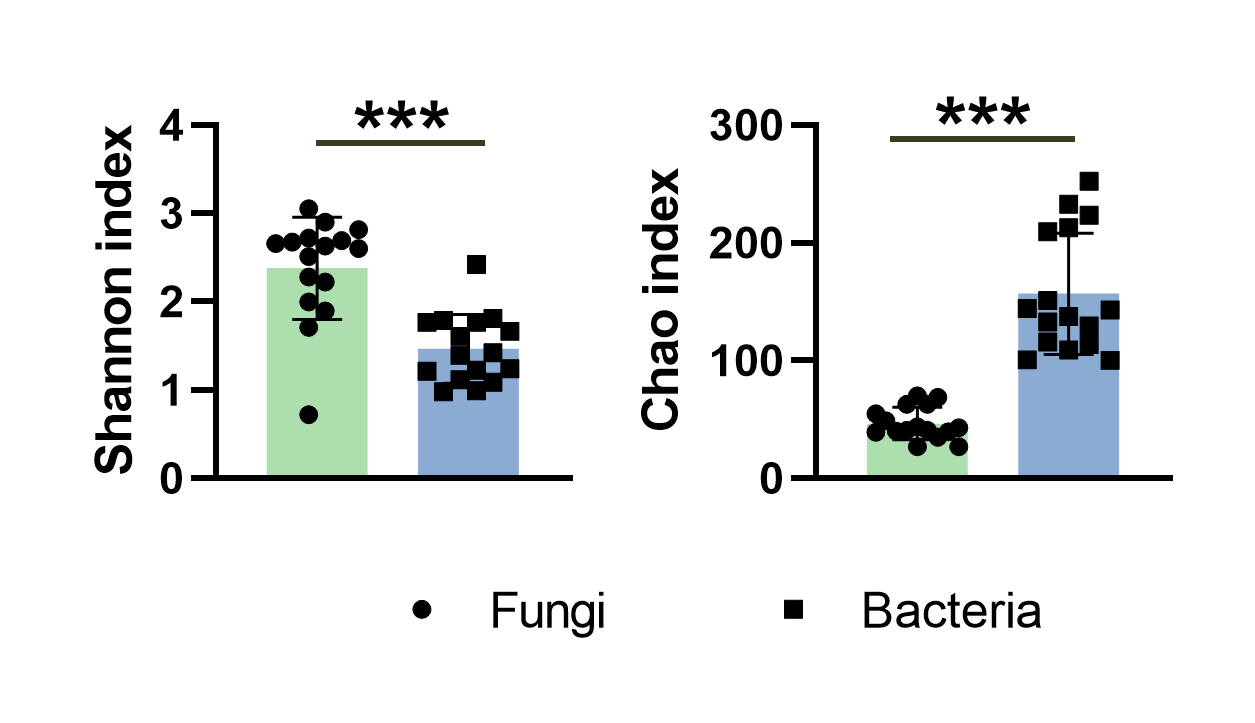


**Gut microbiota of *Folsomia candida***


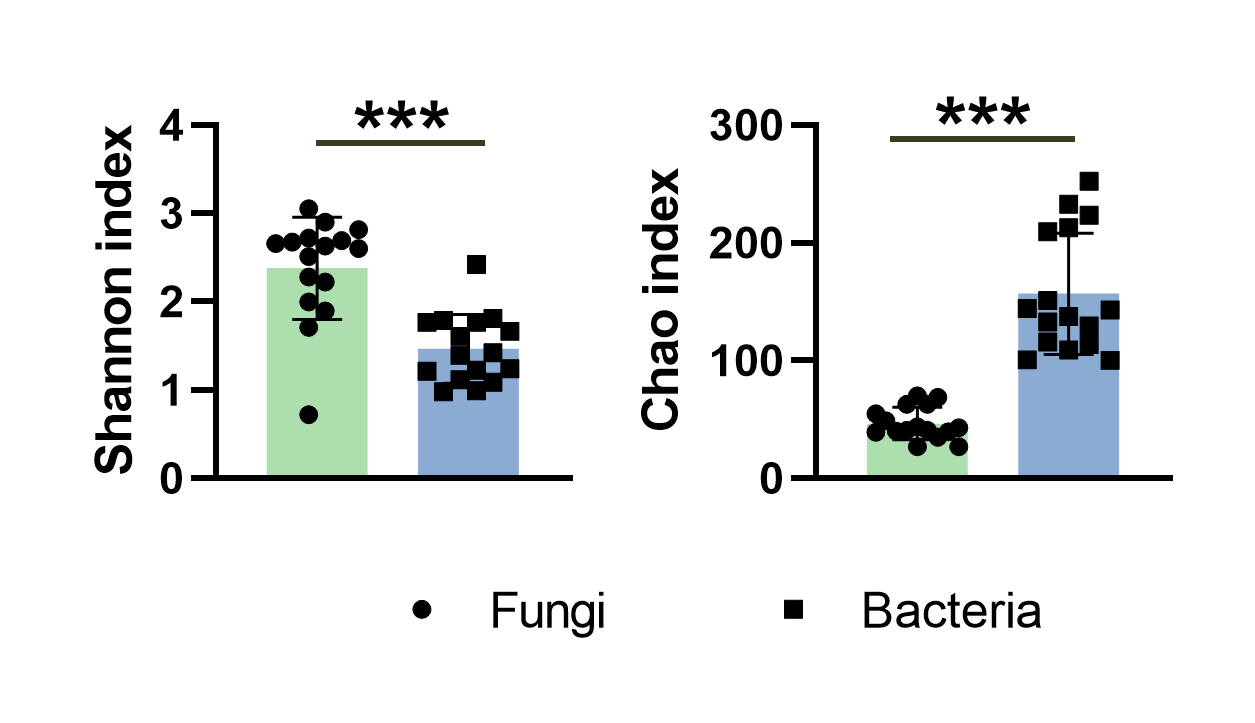

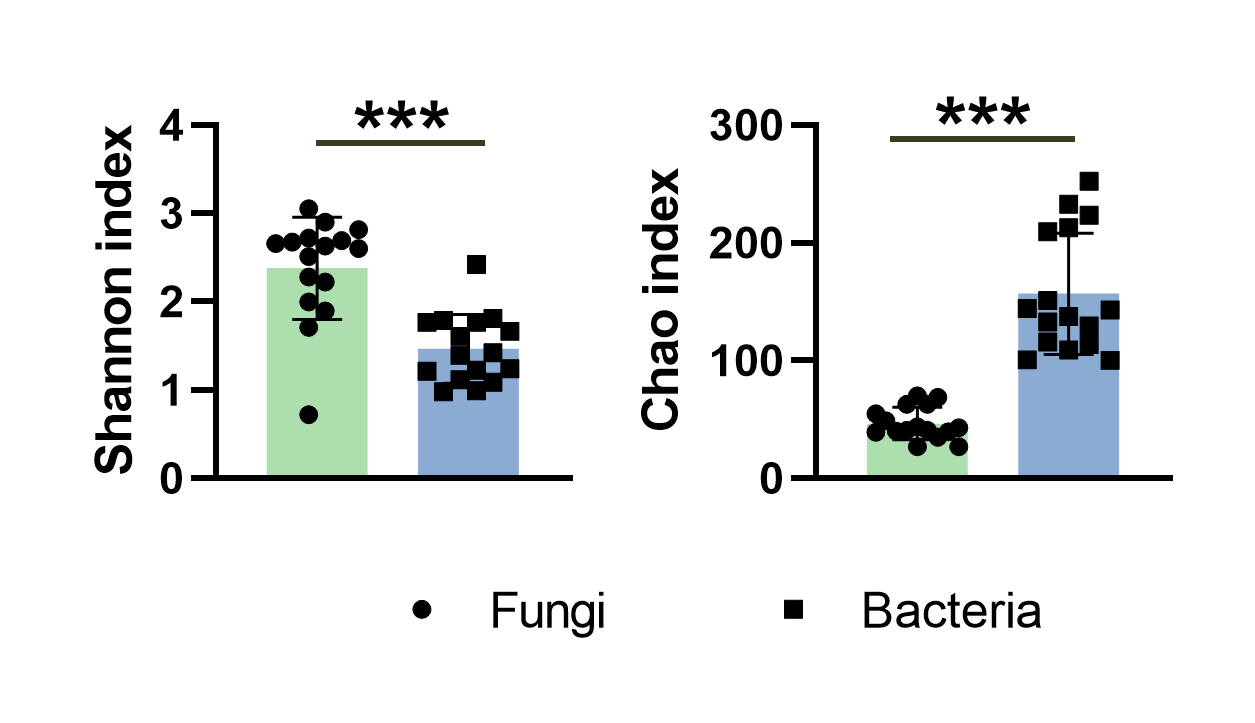


**Figure S5.** Richness (Chao index) of the bacterial and fungal communities in the gut of *Folsomia candida*. *** (*P* < 0.001) indicates significant differences between the bacterial and fungal communities (two-tailed Welch’s *t*-test).


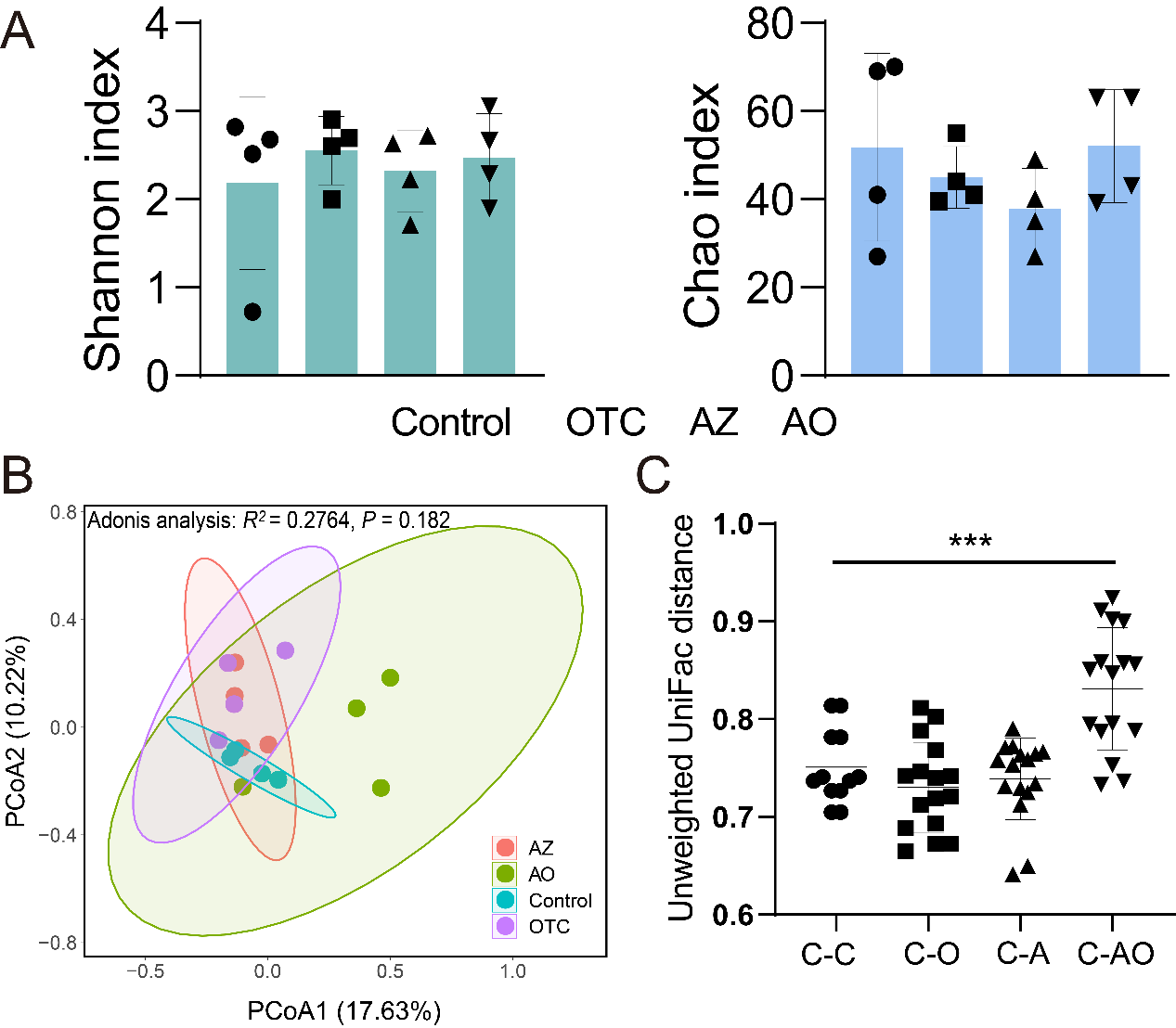


**Figure S6.** Alpha diversity (Shannon and Chao indexes) of the fungal community in the gut of *Folsomia candida* after exposure to 0.3 mg azoxystrobin (AZ)/kg dry soil, 10 mg oxytetracycline (OTC)/kg dry soil, and 0.3 mg AZ + 10 mg OTC/kg dry soil (AO) (**A**). A principal coordinate analysis (PCoA) using unw­eighted UniFrac distances based on the relative abundance of fungal OTUs was performed to account for the differences in the patterns of distribution of the OTUs from the gut of *F. candida* (**B**, **C**). (C-C, C-O, C-A, and C-AO are the distance between control with control, OTC, AZ, and AO, respectively). *** (*P* < 0.001) indicates significant differences between the bacterial and fungal communities (two-tailed Welch’s *t*-test).


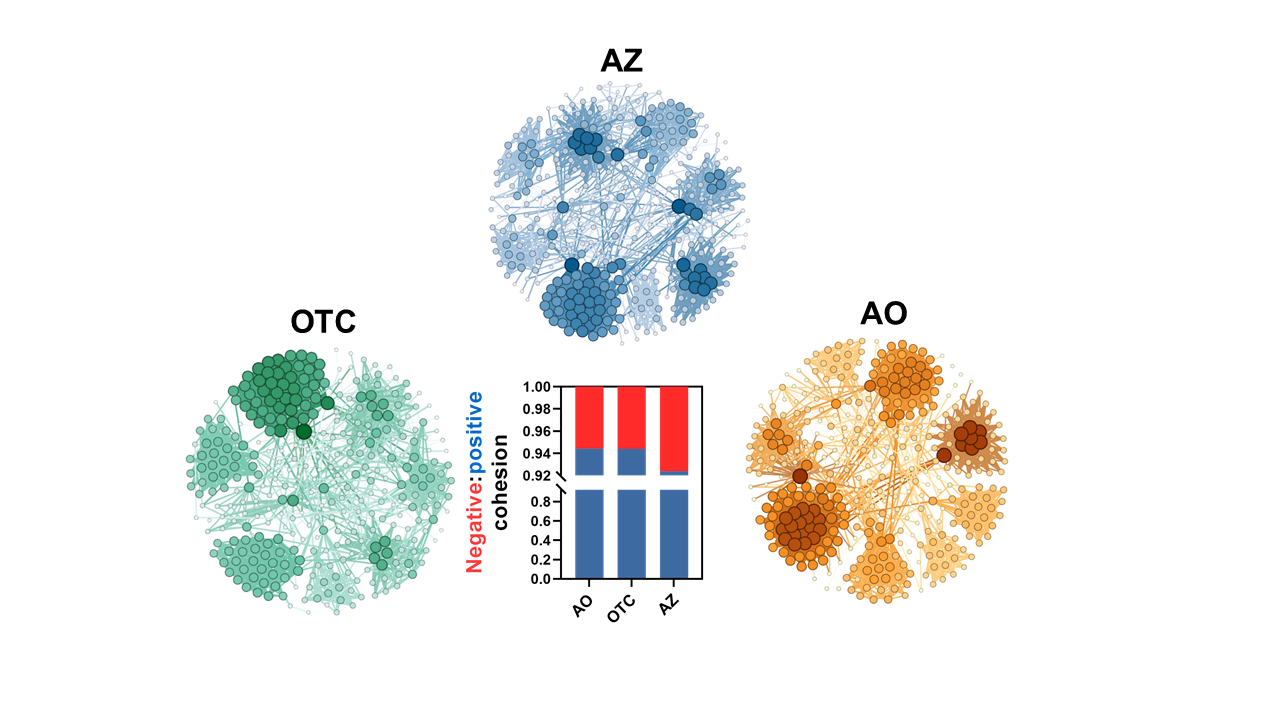


**Figure S7.** Stability of the networks of interaction between bacteria and fungi in the OTC, AZ, and AO groups (*negative*:*positive* cohesion; Pearson analysis, *R^2^* > 0.6, *P* < 0.05).


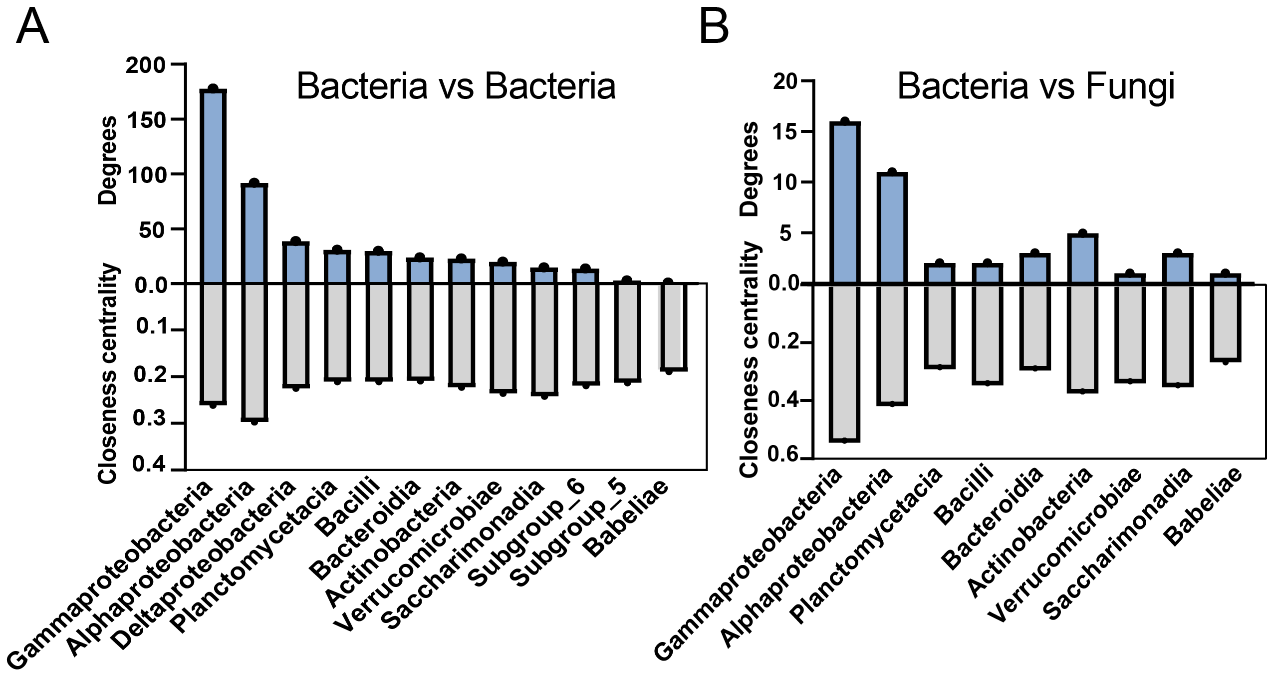


**Figure S8.** The degrees and closeness centrality of bacterial classes (relative abundance) in bacteria-bacteria and bacteria-fungi co-occurrence network from all laboratory samples.


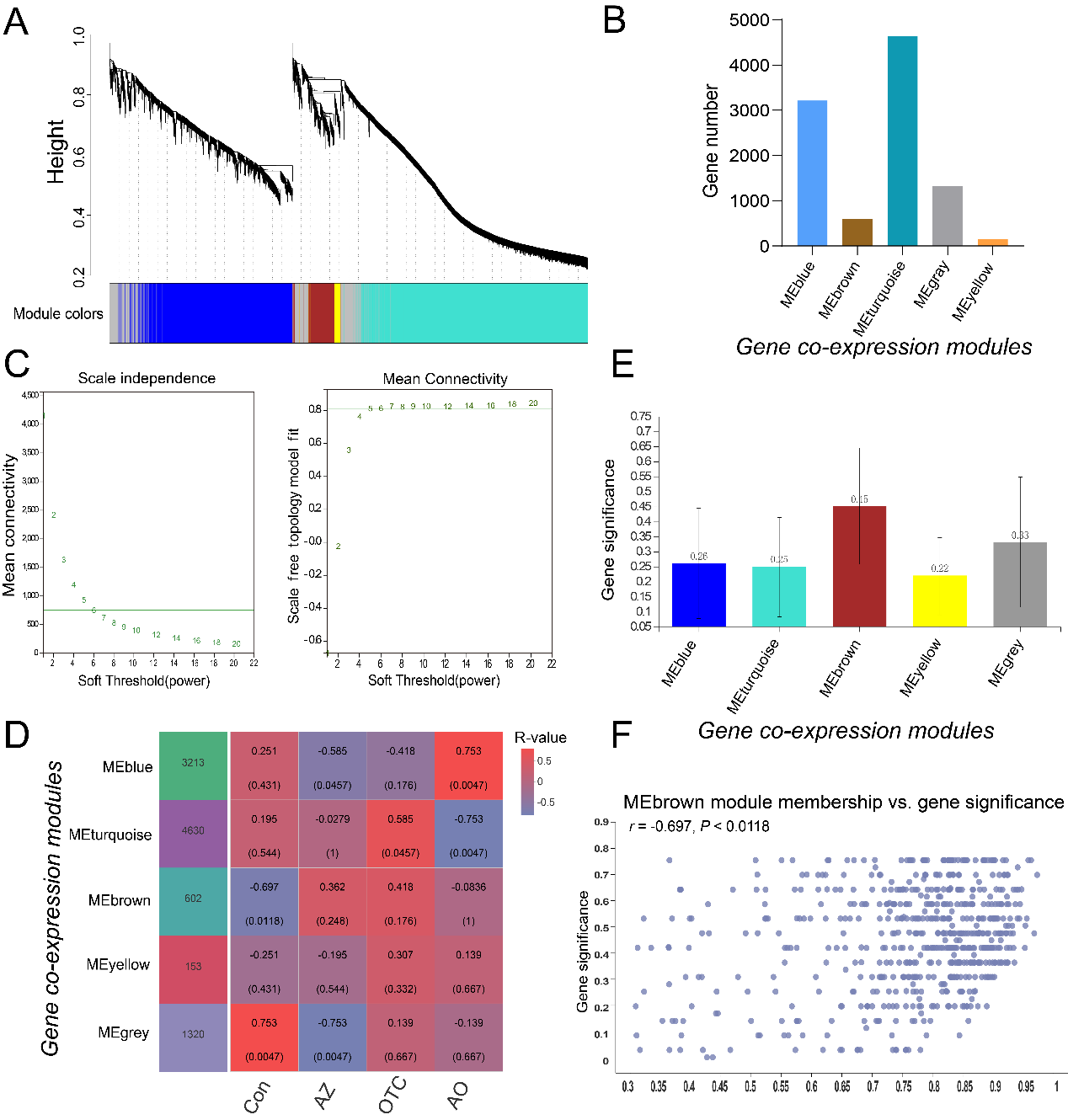


**Figure S9.** WGCNA analysis of *Folsomia candida*. Functional modules are illustrated with five colors (**A**), and gene numbers are shown in **B**. To ensure that the average connectivity of the network was smooth, we chose β = 6 based on both charts: for the topology fitting results and b for mean connectivity (**C**). A strong correlation between a module and a developmental stage is indicated in dark red or dark blue (**D**), each row corresponds to a module, and each column corresponds to a group. The color of each cell at the row-column intersection indicates the correlation coefficient between the modules and the groups. Module significance and correlations significance between MEbrown genes are shown in **E** and **F**. Error bars are the standard error of the total gene significance in different gene co-expression modules. ME colors indicate different gene co-expression modules.


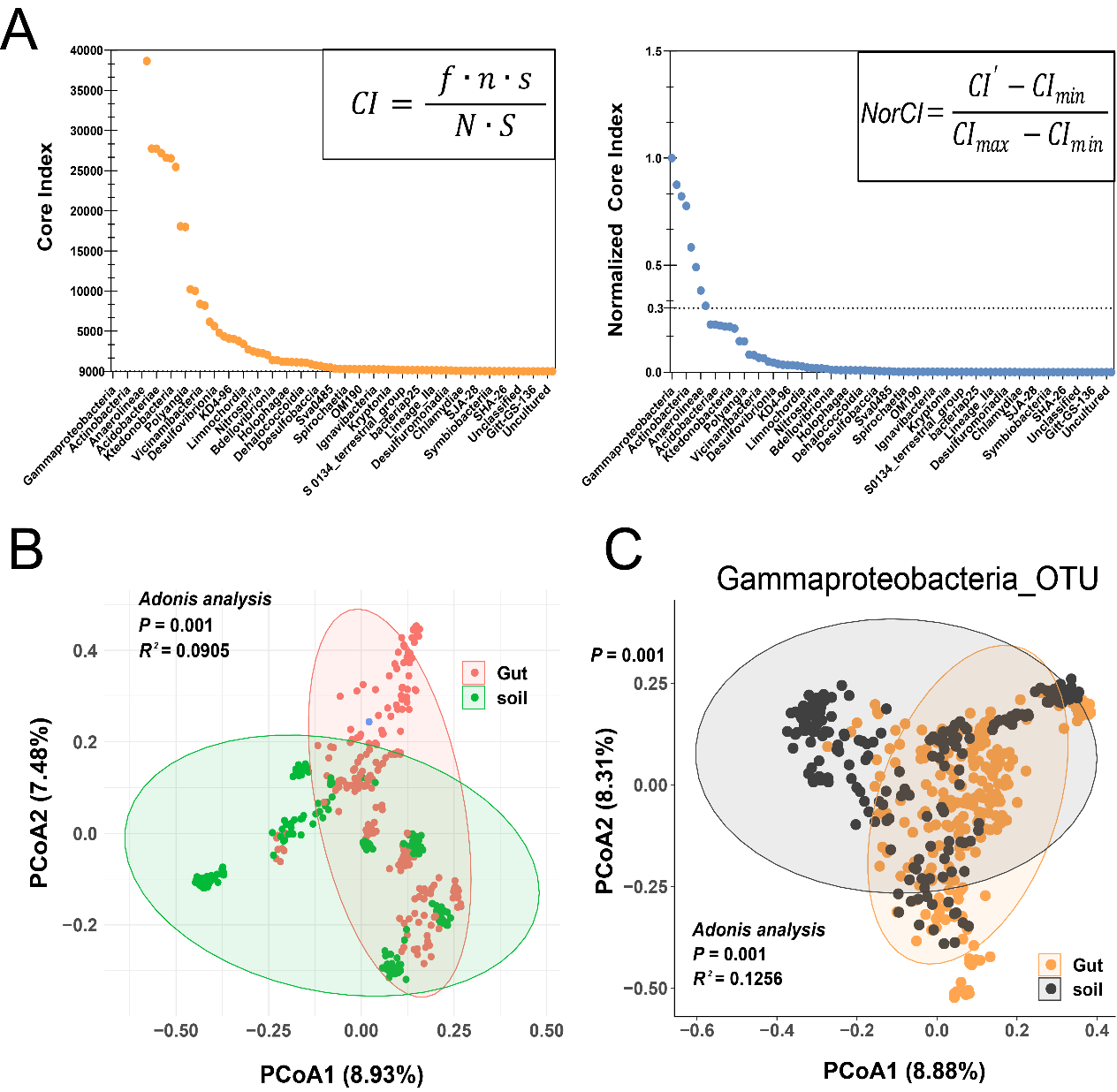


**Figure S10. A:** Core index (CI) and normalized CI (CI’) of the bacterial communities in the soil surrounding the soil invertebrates across eight independent experiments. **B** and **C**: Principal co-ordinate analysis (PCoA) based on the OTU data files using Bray-Curtis distances to characterize all taxa in the soil communities and the Gammaproteobacteria communities in the guts of the soil invertebrates and surrounding soil, respectively. A multivariate PERMANOVA was used to identify significant differences in the bacterial communities between the groups.


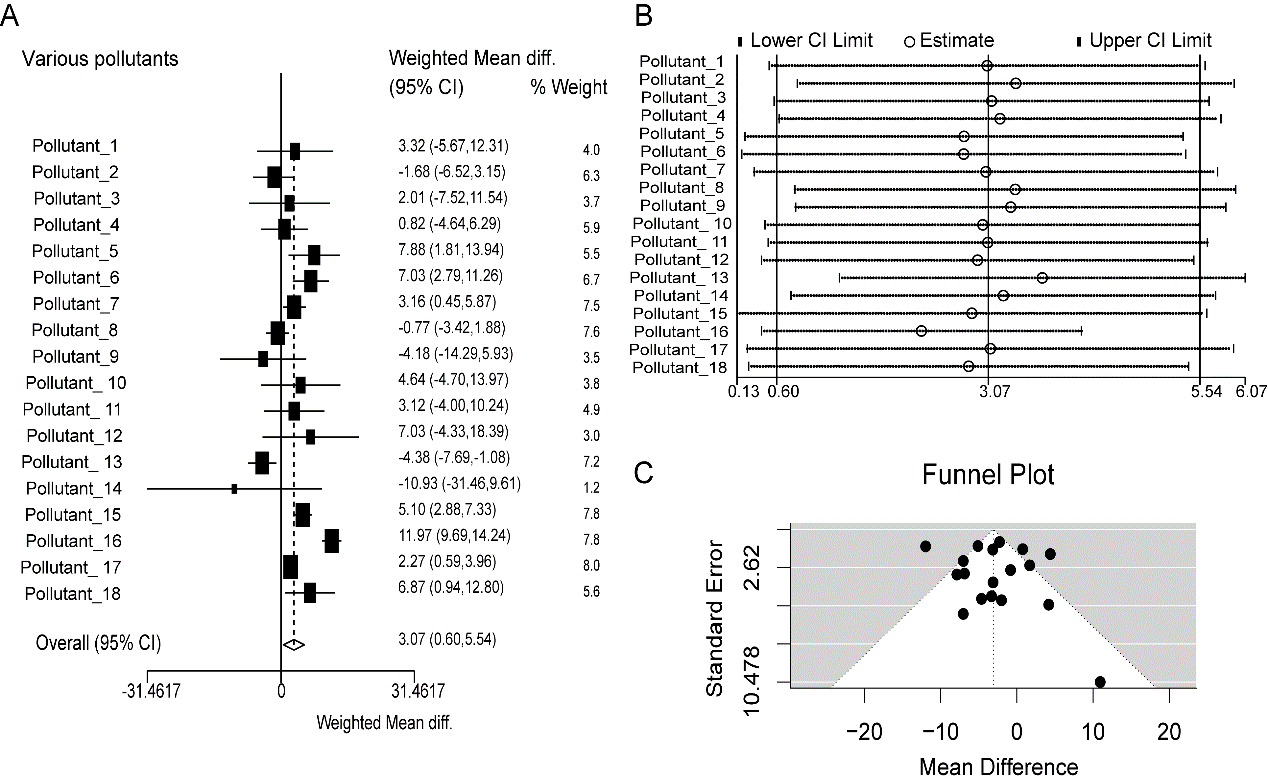


**Figure S11. Meta-analysis and sensitivity analysis of Gammaproteobacteria relative abundance in soil invertebrate guts.** Forest plot estimates for the difference between 18 treatments and control group across the 17 studies (**A**), and the associated 95% CI values (B). Funnel Plot assesses the potential role of publication bias in all independent studies (C).


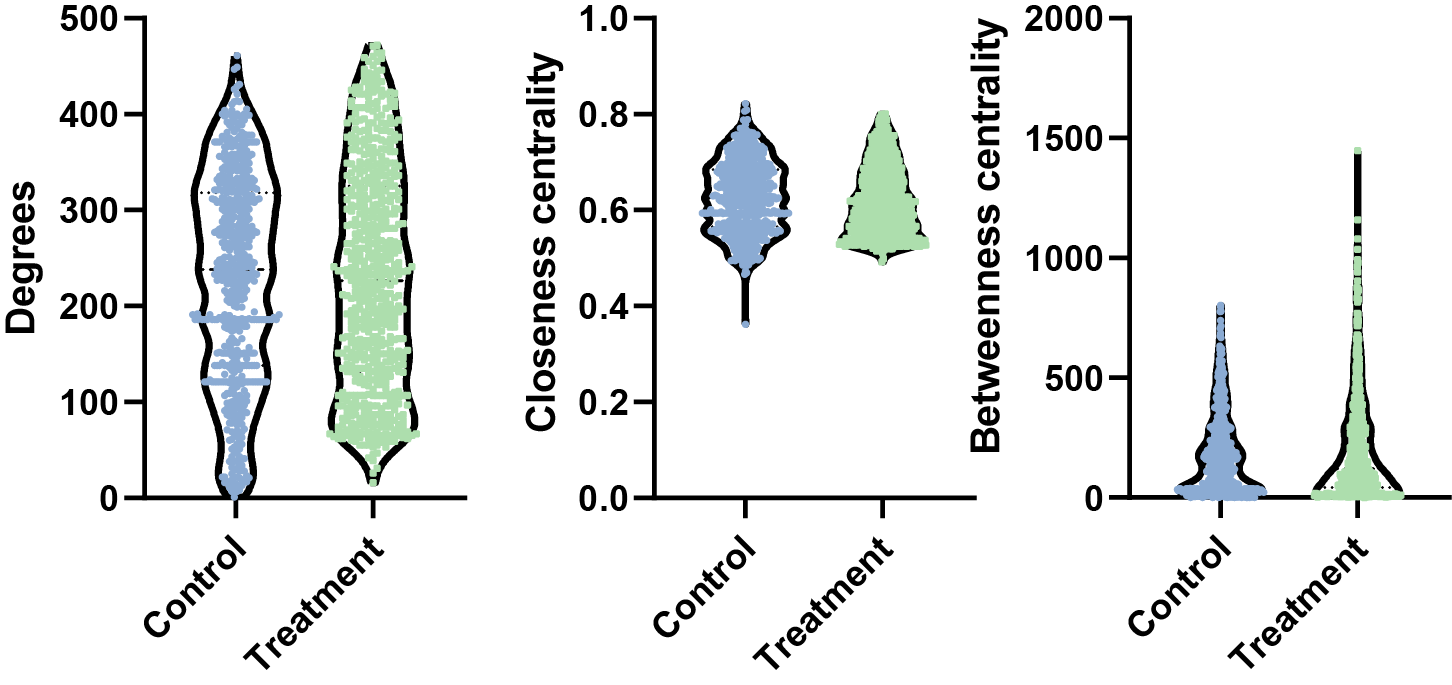


**Figure S12.** The topological properties (degrees, closeness centrality, and betweenness centrality) of bacterial classes in co-occurrence network of control and pollution group from all metadata samples. “ns” indicates no significant differences between the control and treatment group (two-tailed Welch’s *t*-test).


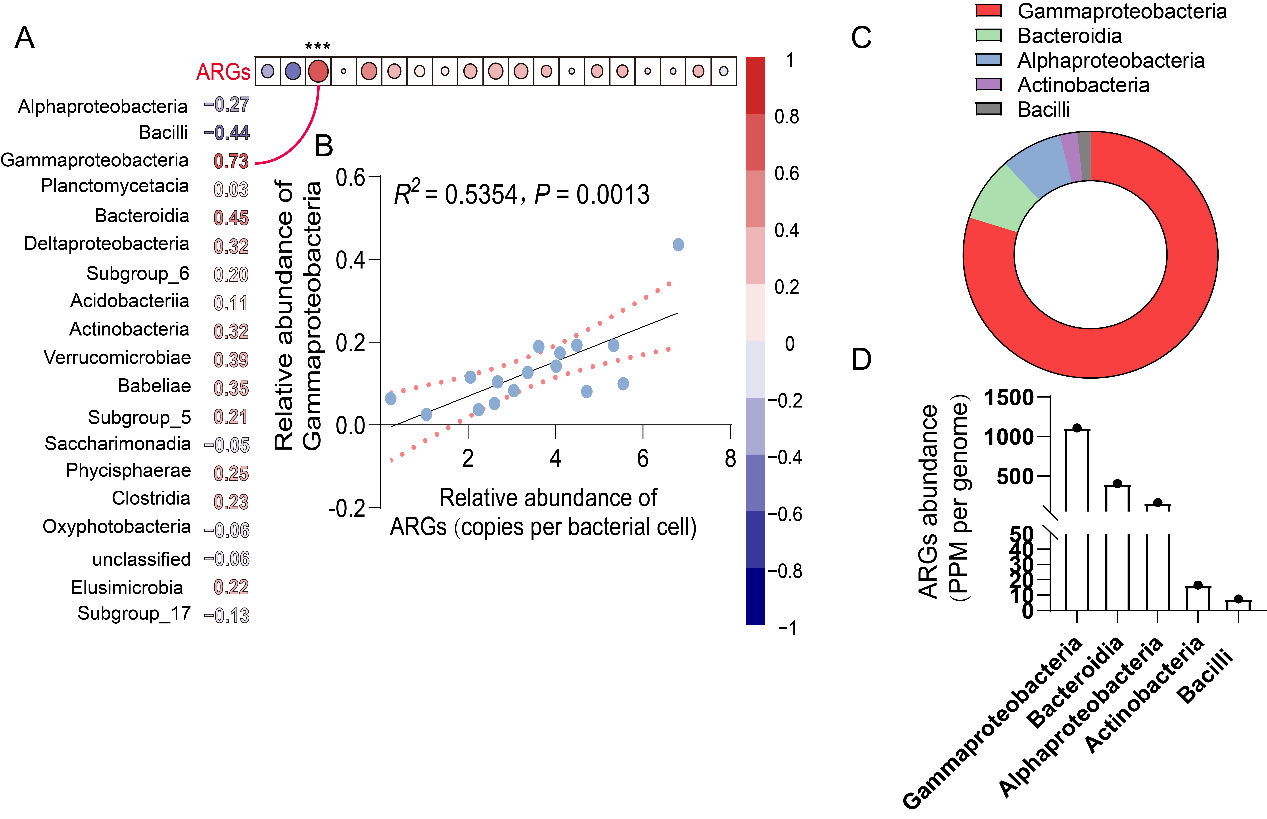


**Figure S13.** Heatmap shows the correlation between bacteria (at class level) and antibiotic resistance genes (ARGs) in *Folsomia candida* gut. “***” indicates the significant correlation (Spearman analysis, *P* < 0.001) (**A**). Ordinary least squares (OLS) linear regression exhibits the correlation-ship between Gammaproteobacteria and ARGs (**B**). Dashed lines indicate the 95% confidence interval.


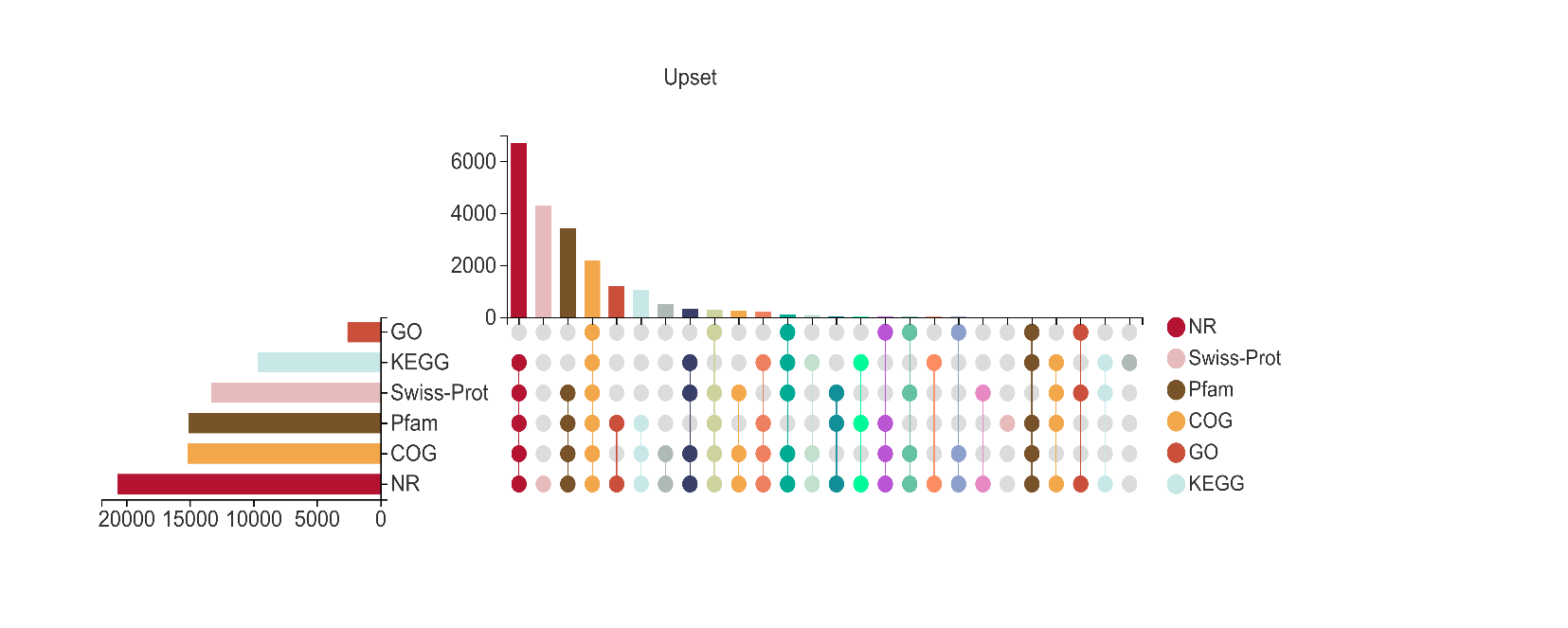


**Figure S14. Annotation of all mapped genes using the GO, KEGG, COG, NR, Swiss-Prot and Pfam databases**. The horizontal histogram on the left represents the statistical values of the elements of each set. Each point in the matrix represents a unique element of a set. The lines between the points represent unique intersections of different sets. The vertical histogram represents the corresponding values of the intersecting elements.

**References**

1. Fountain MT, Hopkin SP: *Folsomia candida* (Collembola): a "standard" soil arthropod. *Annu rev entomol*. 2005;50:201-222.

2. Snyman MG, Naidoo V, de Bruin C, Swan GE: Is the current dose of a conventional oxytetracycline formulation adequate for the management of infections in sheep? *J S Afr Vet Assoc*. 2008; 79(4):171-174.

3. Zhang D, Lin L, Luo Z, Yan C, Zhang X: Occurrence of selected antibiotics in Jiulongjiang River in various seasons, South China. *J Environ monitor*. 2011; 13(7):1953-1960.

4. Ma T, Zhou L, Chen Lk, Li Z, Wu L, Christie P, Luo Y: Oxytetracycline toxicity and its effect on phytoremediation by sedum plumbizincicola and medicago sativa in metal contaminated soil. *J Agrl Food Chem*. 2016;64.

5. Kuppusamy S, Kakarla D, Kadiyala V, Mallavarapu M, Yoon Y, Lee SS: Veterinary antibiotics (VAs) contamination as a global agro-ecological issue: A critical view. *Agr Ecosyst Environ*. 2018; 257:47-59.

6. Adetutu E, Ball A, Osborn A: Azoxystrobin and soil interactions: Degradation and impact on soil bacterial and fungal communities. *J Appl Microbiol*. 2008; 105:1777-1790.

7. Gajbhiye VT, Gupta S, Mukherjee I, Singh SB, Singh N, Dureja P, Kumar Y: Persistence of azoxystrobin in/on grapes and soil in different grapes growing areas of India. *B Environ Contam Tox*. 2011; 86(1):90-94.

8. Liu Y, Sun HB, Zeng FJ, Liu JM: Study on residual dynamics of azoxystrobin in mango and soil. *Guangdong Agricultural Science*. 2010; 10:106-107.

9. Zhang Q, Zhu D, Ding J, Zheng F, Zhou S, Lu T, Zhu Y-G, Qian H: The fungicide azoxystrobin perturbs the gut microbiota community and enriches antibiotic resistance genes in *Enchytraeus crypticus*. *Environ Int*. 2019; 131:104965.

10. Adams RI, Miletto M, Taylor JW, Bruns TD: Dispersal in microbes: fungi in indoor air are dominated by outdoor air and show dispersal limitation at short distances. *The ISME Journal*. 2013; 7(7):1262-1273.

11. Magoč T, Salzberg SL: FLASH: fast length adjustment of short reads to improve genome assemblies. *Bioinformatics*. 2011; 27(21):2957-2963.

12. Chen S, Zhou Y, Chen Y, Gu J: fastp : an ultra-fast all-in-one FASTQ preprocessor; 2018.

13. Edgar RC: UPARSE: highly accurate OTU sequences from microbial amplicon reads. *Nat Methods*. 2013; 10(10):996-998.

14. Vilo C, Dong Q: Evaluation of the RDP classifier accuracy using 16S rRNA gene variable regions. *Metagenomics*. 2012; 1.

15. Quast C, Pruesse E, Yilmaz P, Gerken J, Schweer T, Yarza P, Peplies J, Glöckner FO: The SILVA ribosomal RNA gene database project: improved data processing and web-based tools. *Nucleic Acids Res*. 2013; 41:D590-596.

16. Nilsson RH, Larsson KH, Taylor AFS, Bengtsson-Palme J, Jeppesen TS, Schigel D, Kennedy P, Picard K, Glöckner FO, Tedersoo L et al: The UNITE database for molecular identification of fungi: handling dark taxa and parallel taxonomic classifications. *Nucleic Acids Res*. 2019, 47(D1):D259-d264.

17. Wright R, Langille M, Walker T: Food or just a free ride? A meta-analysis reveals the global diversity of the Plastisphere. *The ISME Journal*. 2021;15:789–806.

18. Zhu D, An X-L, Chen Q-L, Yang X-R, Christie P, Ke X, Wu L-H, Zhu Y-G. Antibiotics disturb the microbiome and increase the incidence of resistance genes in the gut of a common soil collembolan. *Environ Sci Tech*. 2018;52(5):3081-3090.

19. Liaw A, Wiener M: Classification and regression by RandomForest. Forest. 2001;23.

20. Yarnold P, Grimm L. Reading and understanding Multivariate Statistics; 1995.

21. Cortes C, Vapnik V. Support-Vector Networks. Mach Learn. 1995;20(3):273-297.

22. Kurogi S, Shigematsu R, Ono K: Properties of direct multi-step ahead prediction of chaotic time series and out-of-bag estimate for model selection. 2014;8835: 421-428.

23. Astudillo-García C, Hermans SM, Stevenson B, Buckley HL, Lear G. Microbial assemblages and bioindicators as proxies for ecosystem health status: potential and limitations. Appl Microbiol Biot. 2019;103(16):6407-6421.

24. Hernandez DJ, David AS, Menges ES, Searcy CA, Afkhami ME: Environmental stress destabilizes microbial networks. The ISME Journal.2021; 5:1722–1734.

1. ^*^ Corresponding author. Email: hfqian@zjut.edu.cn (Haifeng Qian) [↑](#footnote-ref-1)
