# Supplementary material for: Gammaproteobacteria, a core taxon in the guts of soil fauna, are potential responders to environmental concentrations of soil pollutants
Source: Microbiome. 2021 Sep 30;9:196. doi: 10.1186/s40168-021-01150-6 (PMC8485531; doi:10.1186/s40168-021-01150-6)
Supplement: Supplementary file 3 — Additional files 2: Figure S1. Graphic representation of the experiment and analysis method. Figure S2. Number of adults in the control and all treatments. Figure S3. Alpha diversity (Shannon and Chao indexes) and beta diversity of the bacterial and fungal communities in the soil surrounding Folsomia candida. Figure S4. Number of significantly different fungal OTUs in all treatments compared to the control group. Figure S5. Richness (Chao index) of the bacterial and fungal communities in the gut of Folsomia candida. Figure S6. Alpha diversity (Shannon and Chao indexes) and beta diversity of the fungal community in the gut of Folsomia candida. Figure S7. Stability of the networks of interaction between bacteria and fungi in all groups. Figure S8. The degrees and closeness centrality of bacterial classes (relative abundance) in bacteria-bacteria and bacteria-fungi co-occurrence networks from all laboratory samples. Figure S9. WGCNA analysis of Folsomia candida. Figure S10. Core index (CI) and normalized CI (NorCI) of the bacterial communities in the soil surrounding the soil invertebrates across eight independent experiments. Figure S11. Meta-analysis and sensitivity analysis of Gammaproteobacteria relative abundance in soil invertebrate guts. Figure S12. The topological properties (degrees, closeness centrality, and betweenness centrality) of bacterial classes in the co-occurrence networks of control and pollution group from all metadata samples. Figure S13. The identification of antibiotic resistance bacteria (ARB) in soil invertebrate gut. Figure S14. Annotation of all mapped genes using the GO, KEGG, COG, NR, Swiss-Prot, and Pfam databases. [file 40168_2021_1150_MOESM3_ESM.docx]

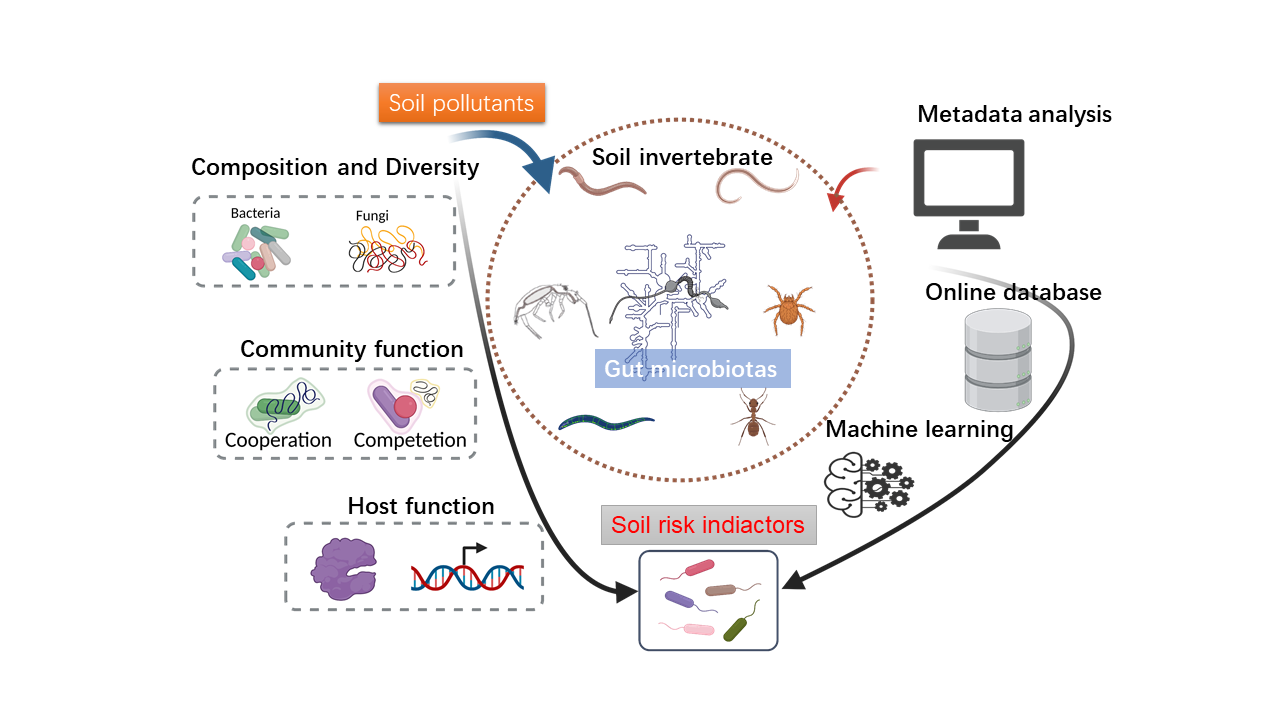


**Figure S1.** Graphic representation of the experiment and analysis method.


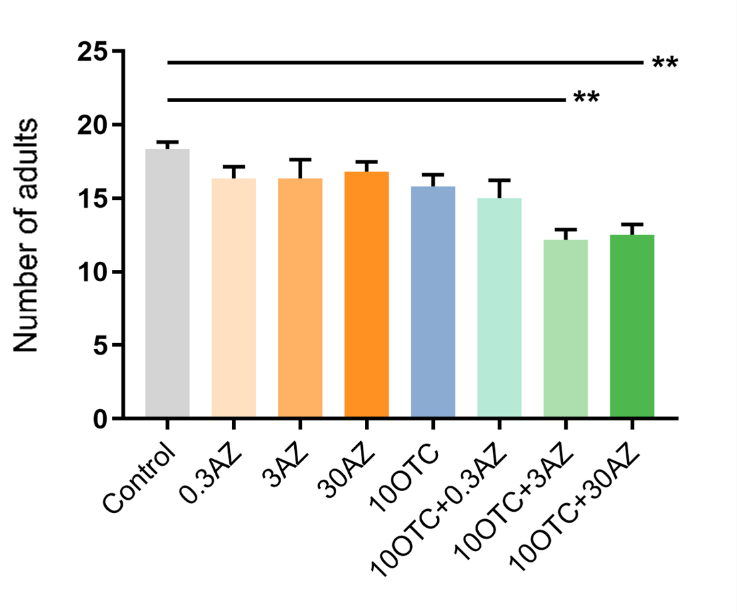


**Figure S2.** Number of adults in the control and the treatments with 0.3, 3, and 30 mg azoxystrobin (AZ)/kg dry soil (0.3AZ, 3AZ, and 30AZ), 10 mg oxytetracycline (OTC)/kg dry soil (10OTC), and 0.3, 3, and 30 mg AZ + 10 mg OTC/kg dry soil (10OTC+0.3AZ, 10OTC+3AZ, and 10OTC+30AZ). ** (*P* < 0.01) indicates significant differences between the control and the treatments (two-tailed Welch’s *t*-test).


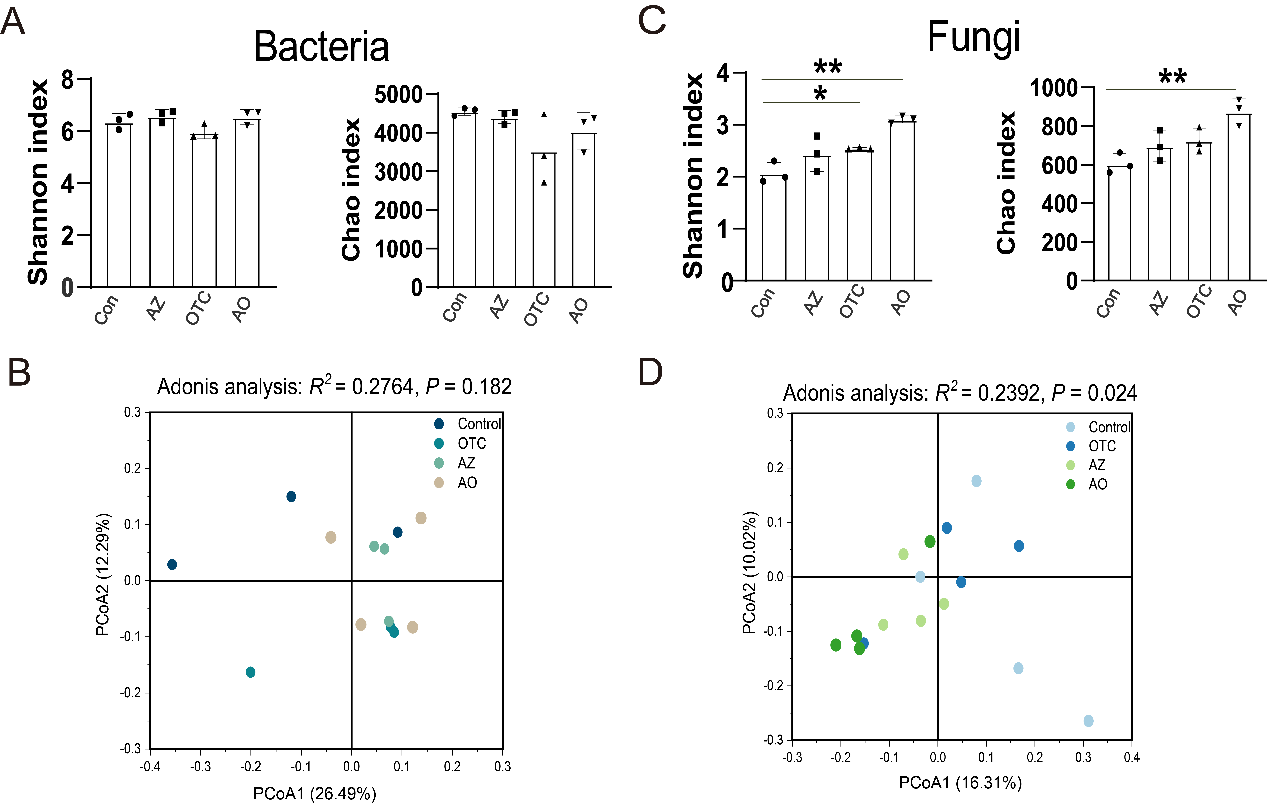


**Figure S3.** Alpha diversity (Shannon and Chao indexes) of the bacterial and fungal communities in the soil surrounding *Folsomia candida* after exposure to 0.3 mg Azoxystrobin (AZ)/kg dry soil, 10 mg oxytetracycline (OTC)/kg dry soil, and 0.3 mg AZ + 10 mg OTC/kg dry soil (AO) (**A**, **C**). A principal coordinate analysis (PCoA) and a multivariate PERMANOVA using unweighted UniFrac distances based on the relative abundances of soil bacterial and fungal OTUs were performed to account for the differences in the patterns of distribution of the OTUs in the surrounding soil (**B**, **D**). “*” and “**” (*P* < 0.05 and *P* < 0.01, respectively) indicate significant differences between the control and the treatments (two-tailed Welch’s *t*-test).


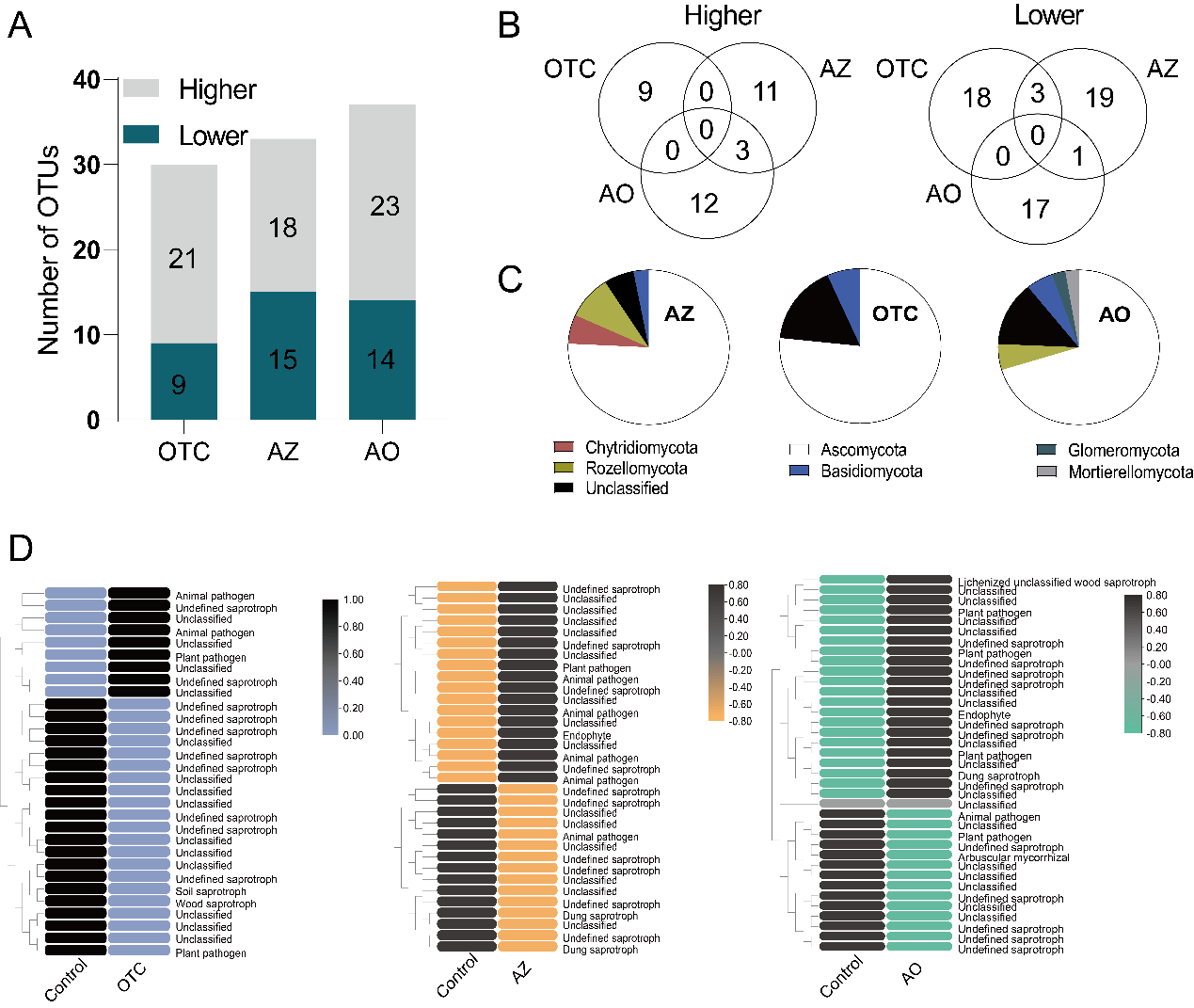


**Figure S4.** Number of significantly different fungal OTUs in the OTC, AZ, and AO groups compared to the control (MetagenomeSeq analysis) (**A**). The gray and green columns indicate OTUs with lower and higher relative abundances, respectively. The numbers and taxa of shared significantly different OTUs among the OTC, AZ, and AO groups (**B**, **C**). Heatmaps of the functional classification of the significantly different fungal OTUs in the OTC, AZ, and AO groups (**D**).


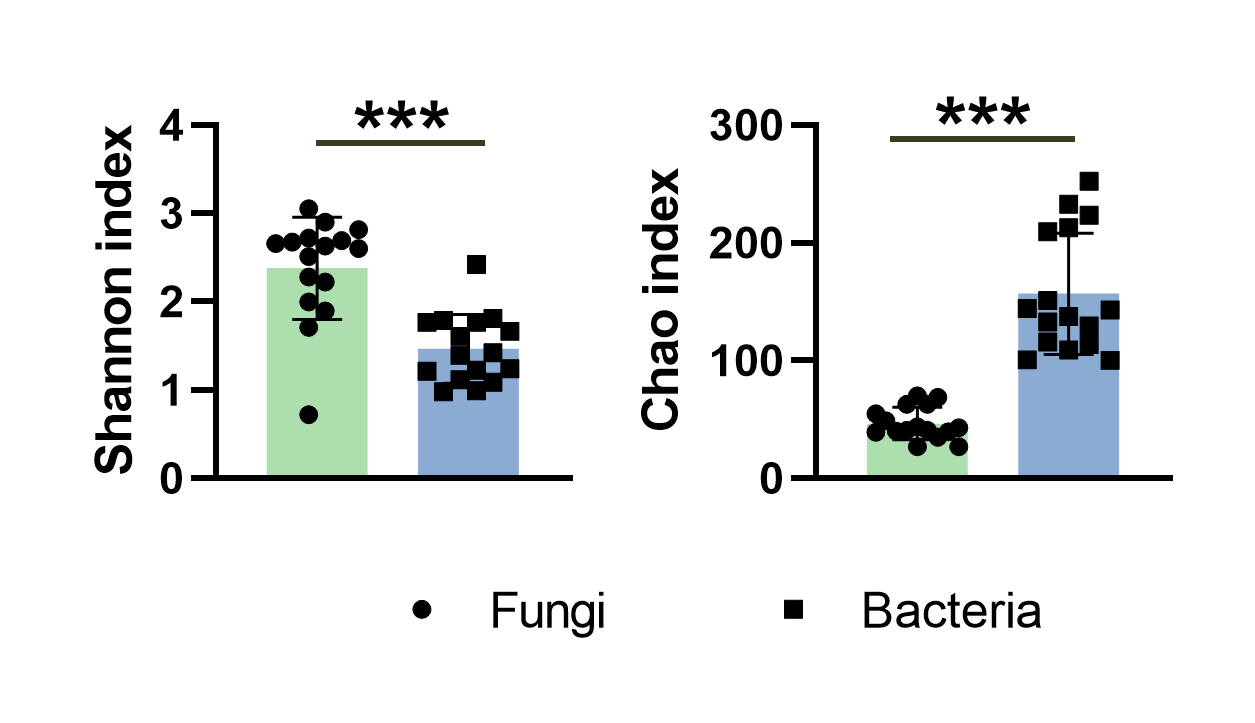


**Gut microbiota of *Folsomia candida***


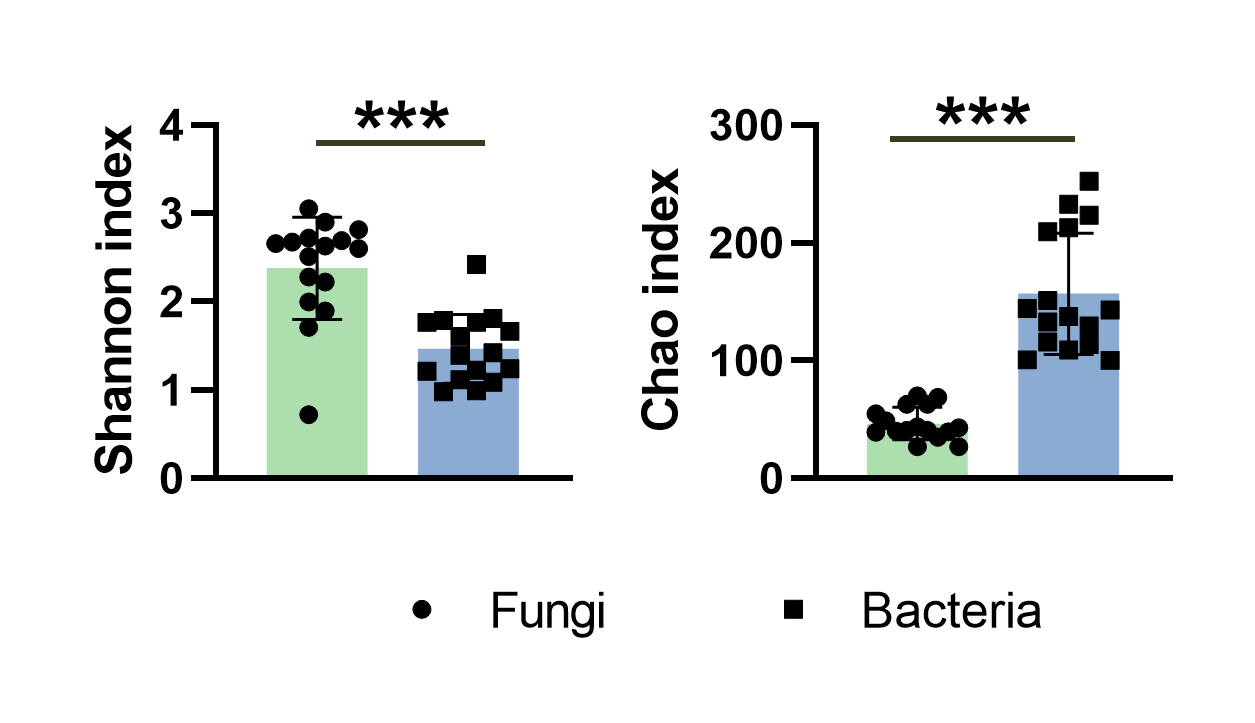

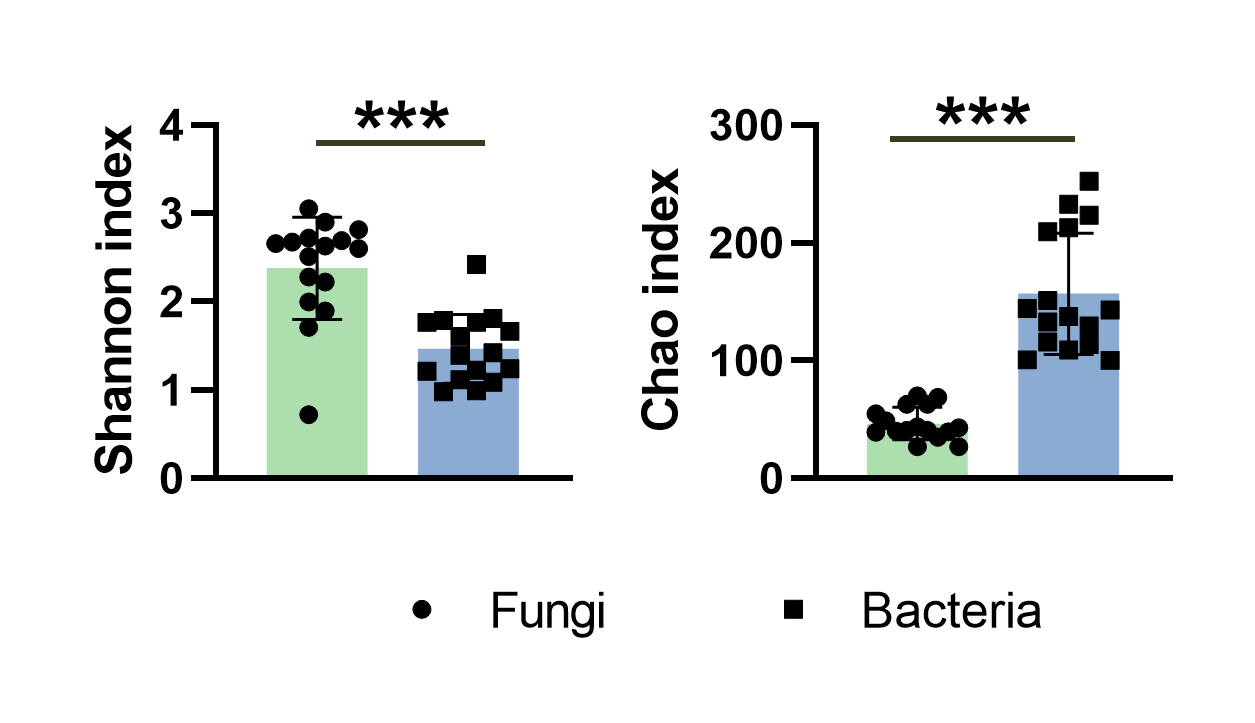


**Figure S5.** Richness (Chao index) of the bacterial and fungal communities in the gut of *Folsomia candida*. *** (*P* < 0.001) indicates significant differences between the bacterial and fungal communities (two-tailed Welch’s *t*-test).


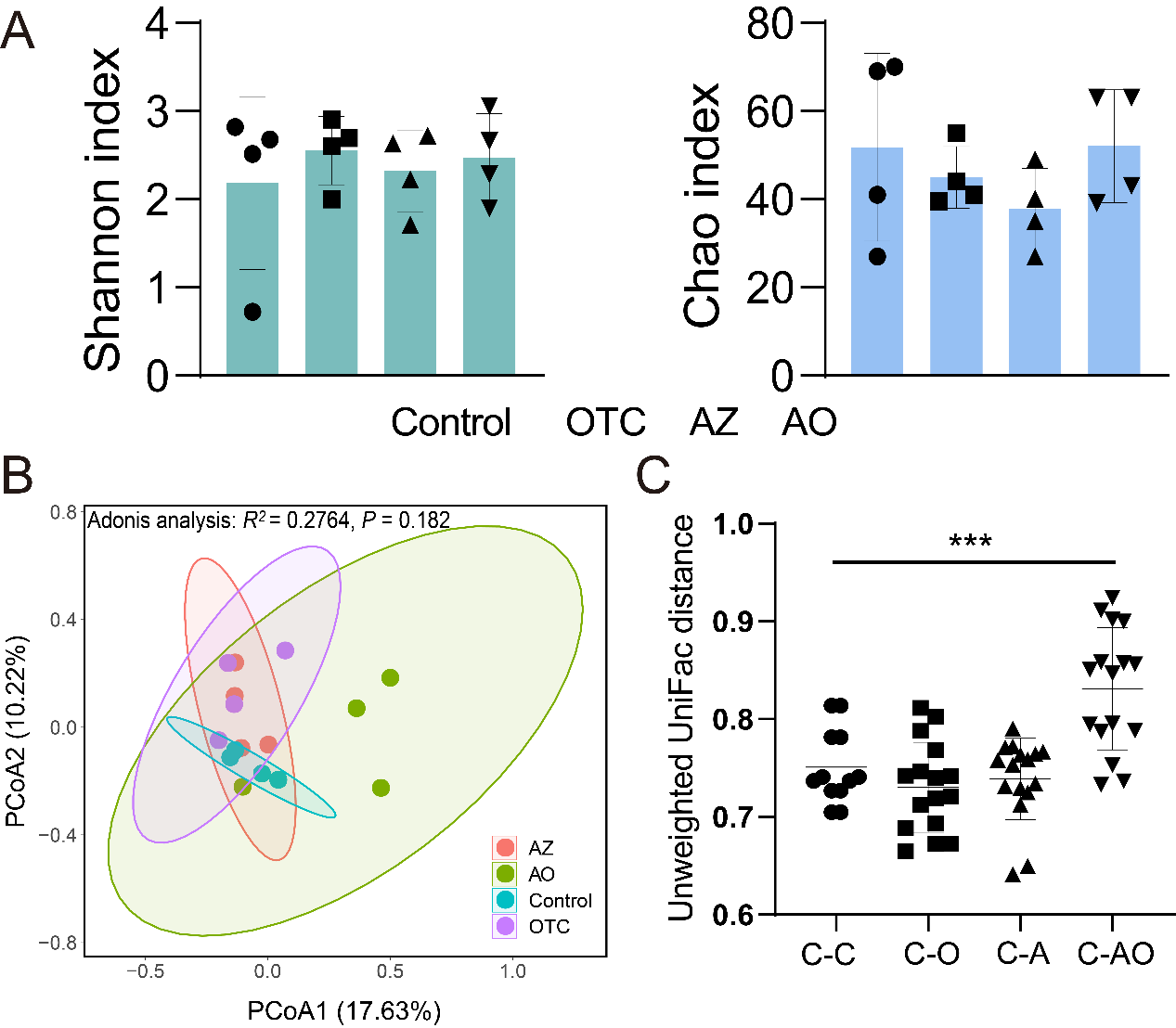


**Figure S6.** Alpha diversity (Shannon and Chao indexes) of the fungal community in the gut of *Folsomia candida* after exposure to 0.3 mg azoxystrobin (AZ)/kg dry soil, 10 mg oxytetracycline (OTC)/kg dry soil, and 0.3 mg AZ + 10 mg OTC/kg dry soil (AO) (**A**). A principal coordinate analysis (PCoA) using unw­eighted UniFrac distances based on the relative abundance of fungal OTUs was performed to account for the differences in the patterns of distribution of the OTUs from the gut of *F. candida* (**B**, **C**). (C-C, C-O, C-A, and C-AO are the distance between control with control, OTC, AZ, and AO, respectively). *** (*P* < 0.001) indicates significant differences between the bacterial and fungal communities (two-tailed Welch’s *t*-test).


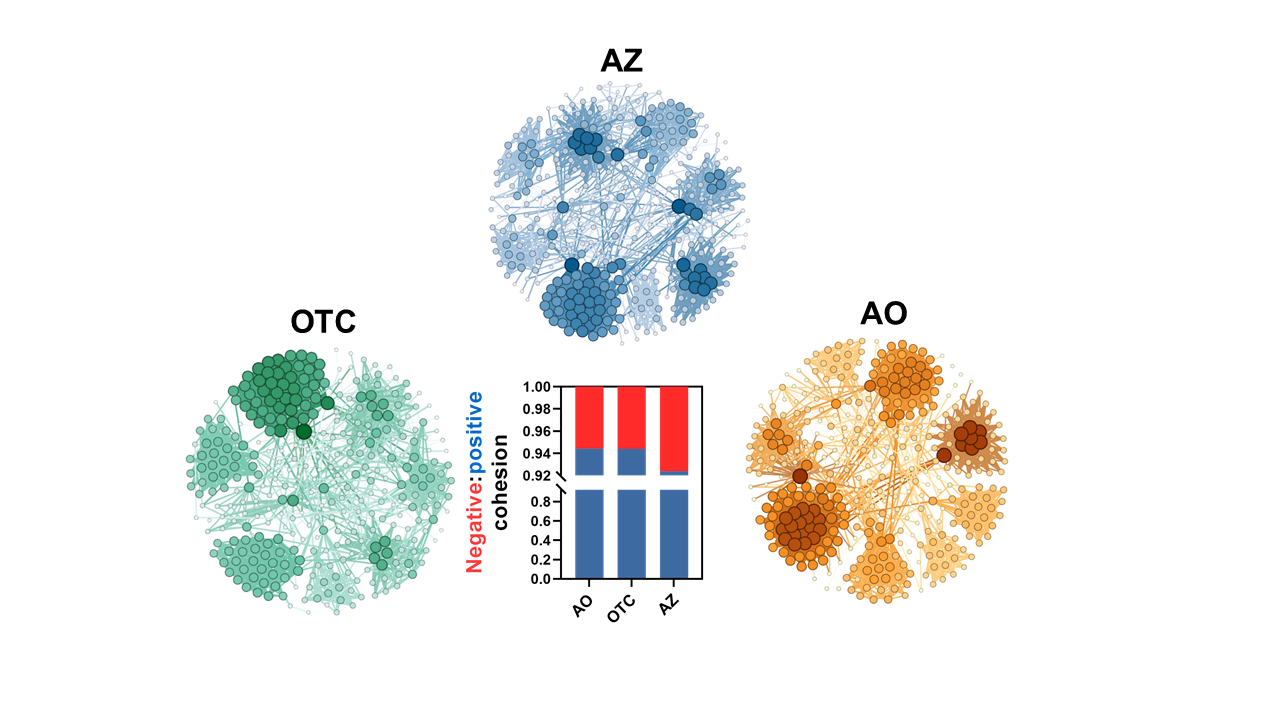


**Figure S7.** Stability of the networks of interaction between bacteria and fungi in the OTC, AZ, and AO groups (*negative*:*positive* cohesion; Pearson analysis, *R^2^* > 0.6, *P* < 0.05).


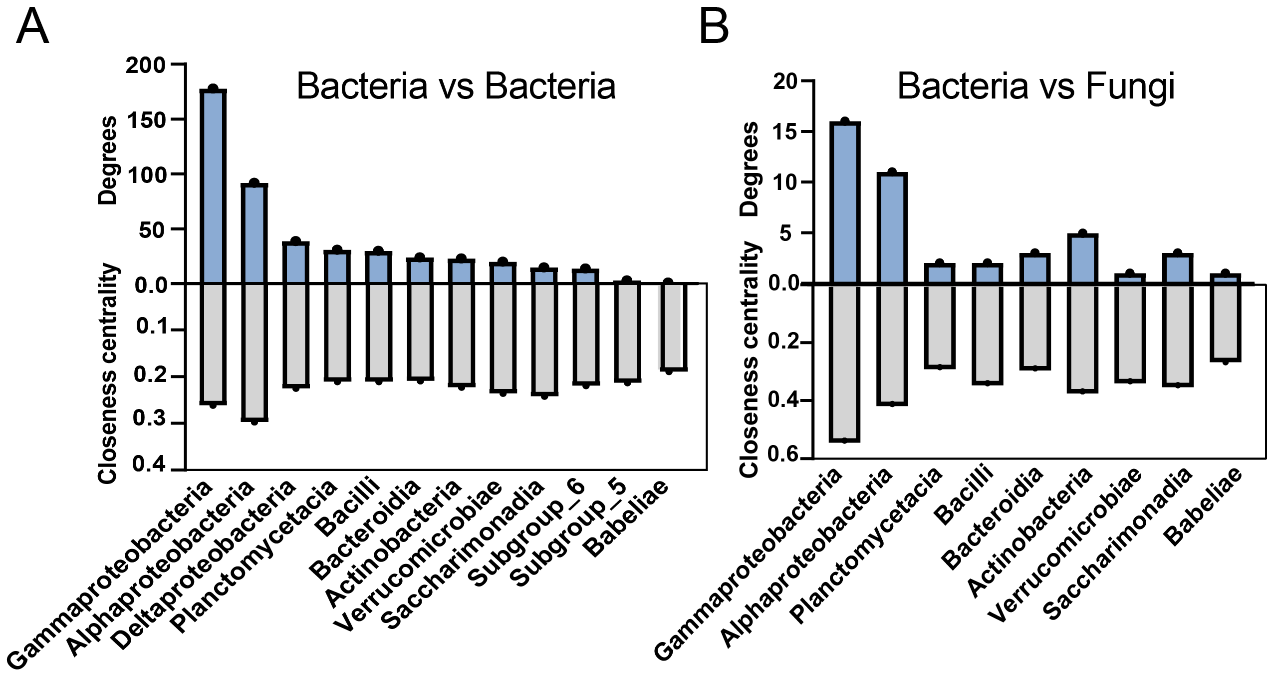


**Figure S8.** The degrees and closeness centrality of bacterial classes (relative abundance) in bacteria-bacteria and bacteria-fungi co-occurrence network from all laboratory samples.


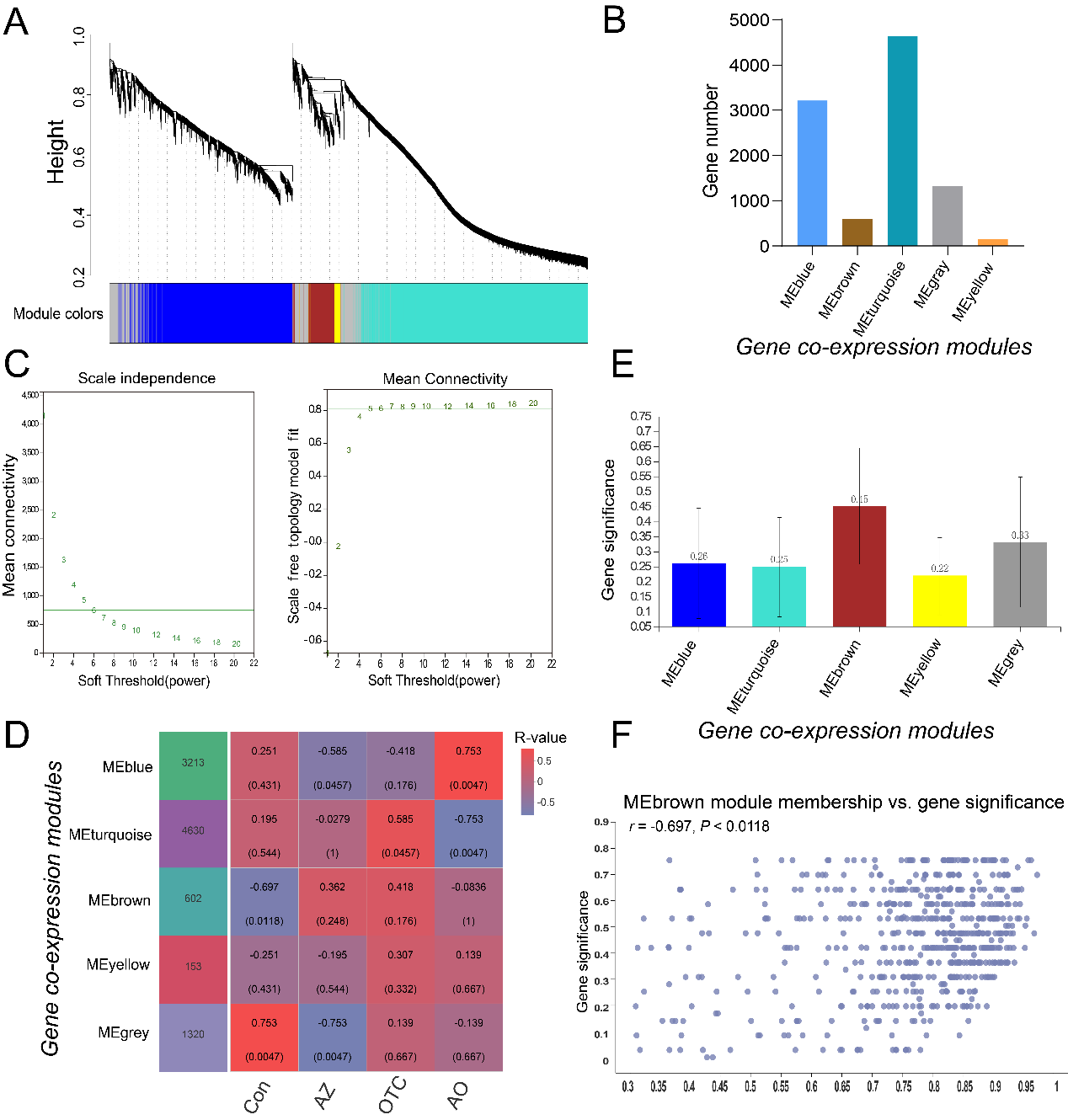


**Figure S9.** WGCNA analysis of *Folsomia candida*. Functional modules are illustrated with five colors (**A**), and gene numbers are shown in **B**. To ensure that the average connectivity of the network was smooth, we chose β = 6 based on both charts: for the topology fitting results and b for mean connectivity (**C**). A strong correlation between a module and a developmental stage is indicated in dark red or dark blue (**D**), each row corresponds to a module, and each column corresponds to a group. The color of each cell at the row-column intersection indicates the correlation coefficient between the modules and the groups. Module significance and correlations significance between MEbrown genes are shown in **E** and **F**. Error bars are the standard error of the total gene significance in different gene co-expression modules. ME colors indicate different gene co-expression modules.


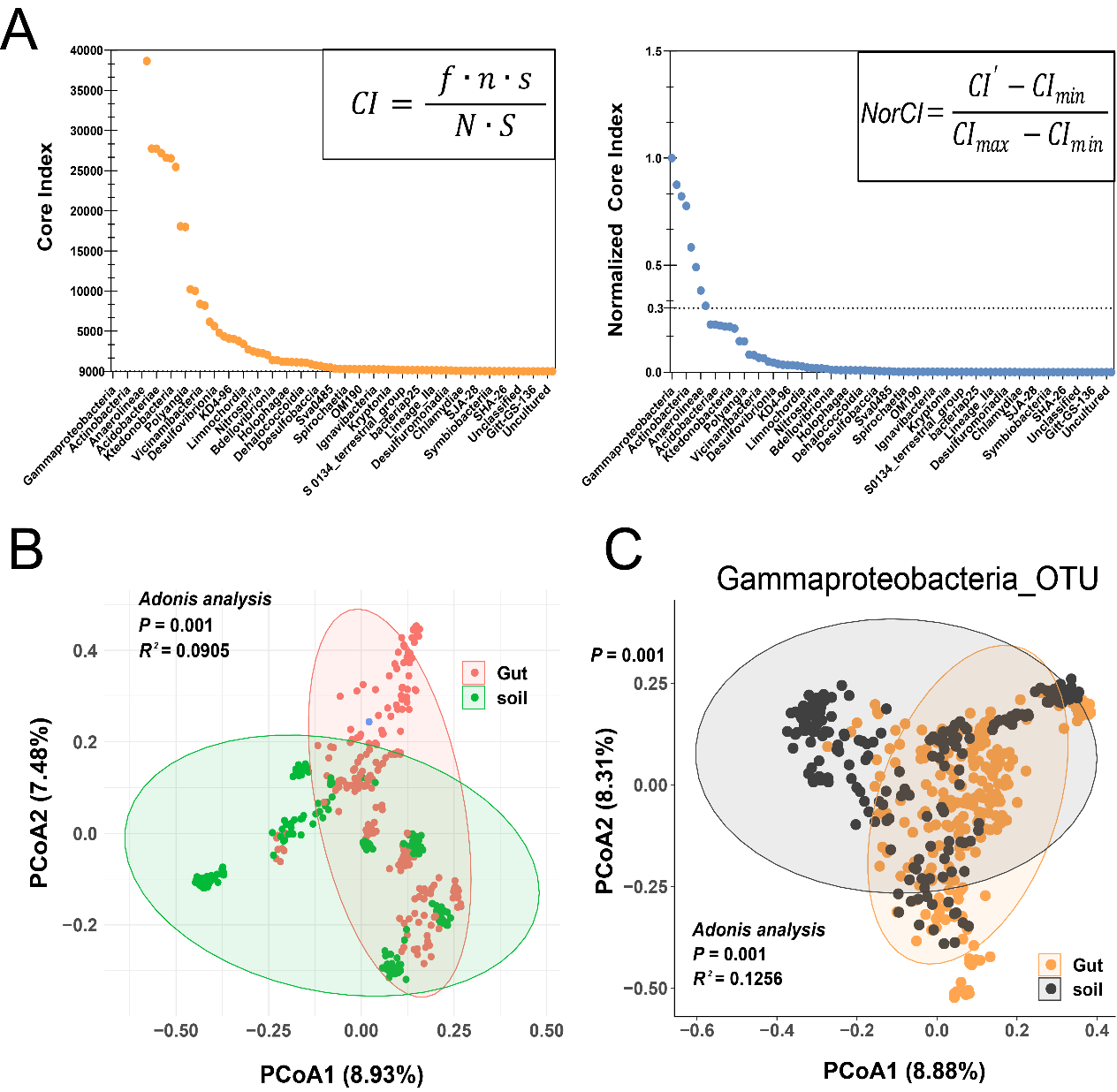


**Figure S10. A:** Core index (CI) and normalized CI (CI’) of the bacterial communities in the soil surrounding the soil invertebrates across eight independent experiments. **B** and **C**: Principal co-ordinate analysis (PCoA) based on the OTU data files using Bray-Curtis distances to characterize all taxa in the soil communities and the Gammaproteobacteria communities in the guts of the soil invertebrates and surrounding soil, respectively. A multivariate PERMANOVA was used to identify significant differences in the bacterial communities between the groups.


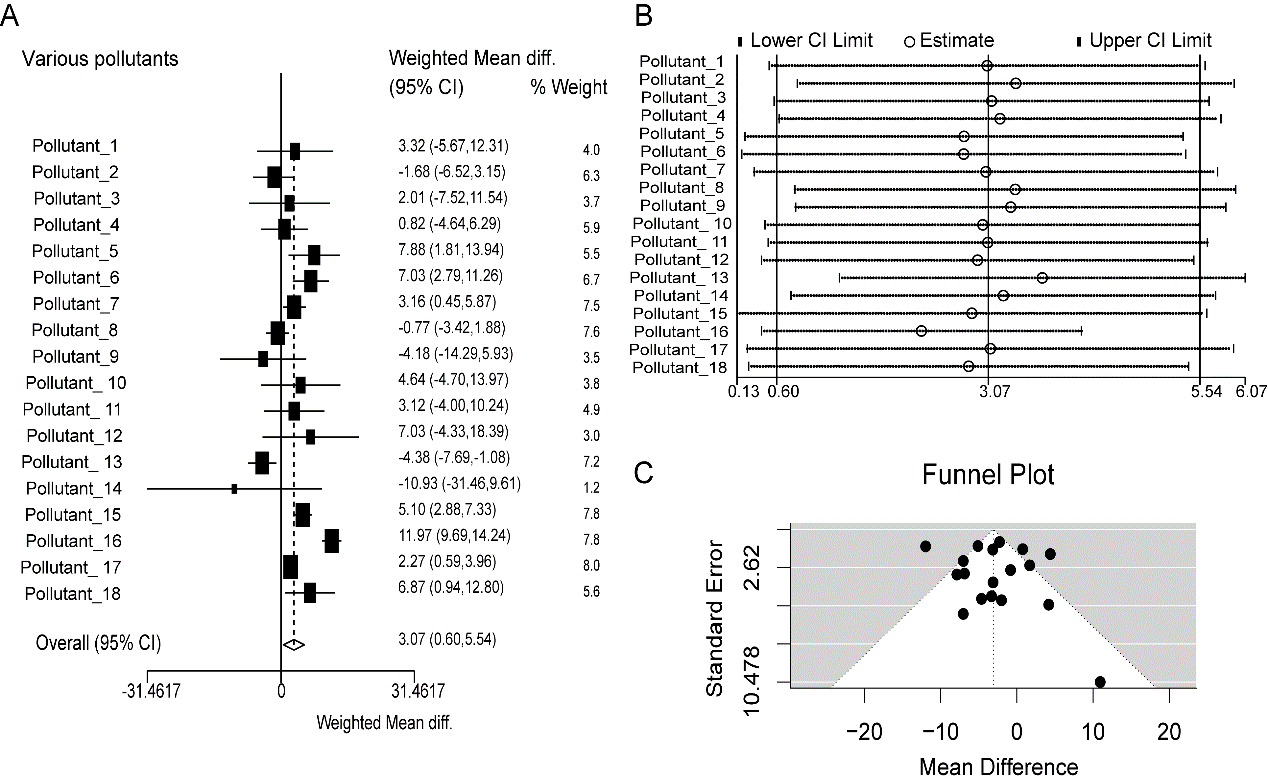


**Figure S11. Meta-analysis and sensitivity analysis of Gammaproteobacteria relative abundance in soil invertebrate guts.** Forest plot estimates for the difference between 18 treatments and control group across the 17 studies (**A**), and the associated 95% CI values (B). Funnel Plot assesses the potential role of publication bias in all independent studies (C).


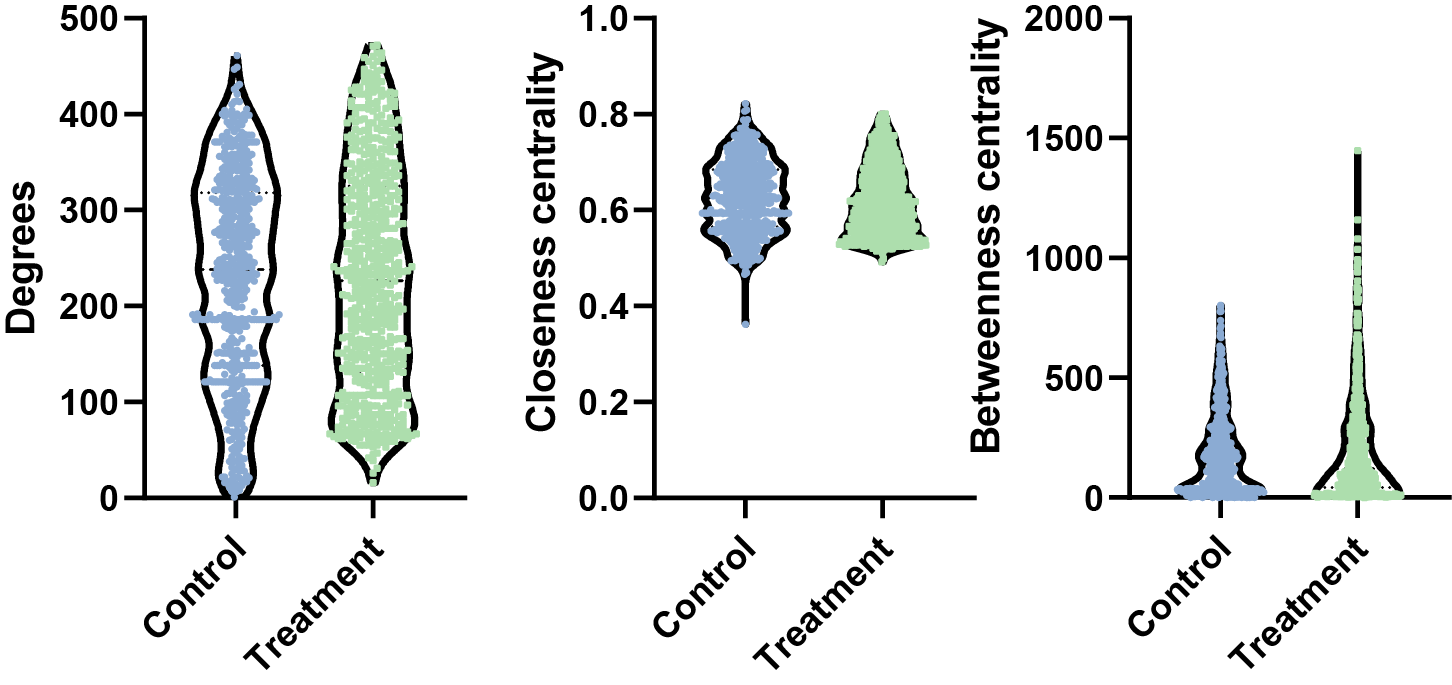


**Figure S12.** The topological properties (degrees, closeness centrality, and betweenness centrality) of bacterial classes in co-occurrence network of control and pollution group from all metadata samples. “ns” indicates no significant differences between the control and treatment group (two-tailed Welch’s *t*-test).


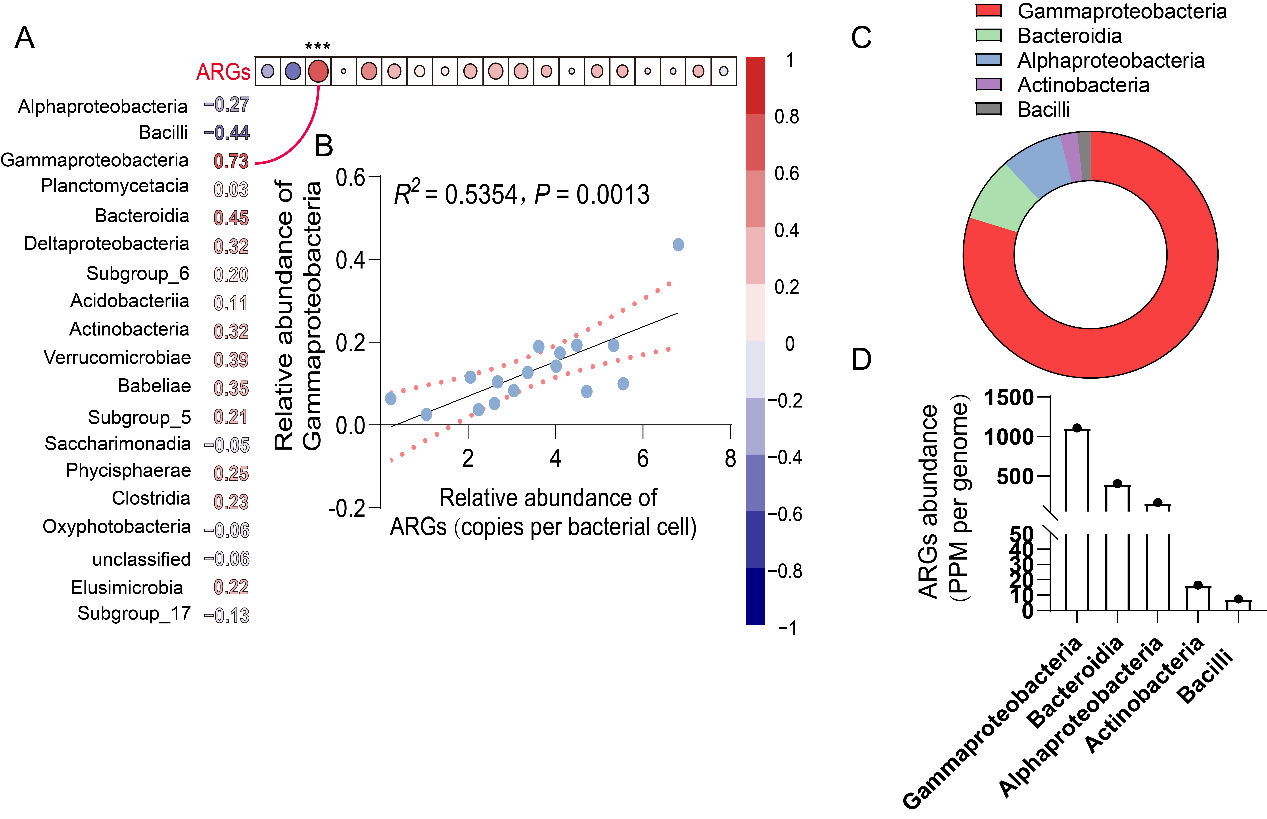


**Figure S13.** Heatmap shows the correlation between bacteria (at class level) and antibiotic resistance genes (ARGs) in *Folsomia candida* gut. “***” indicates the significant correlation (Spearman analysis, *P* < 0.001) (**A**). Ordinary least squares (OLS) linear regression exhibits the correlation-ship between Gammaproteobacteria and ARGs (**B**). Dashed lines indicate the 95% confidence interval.


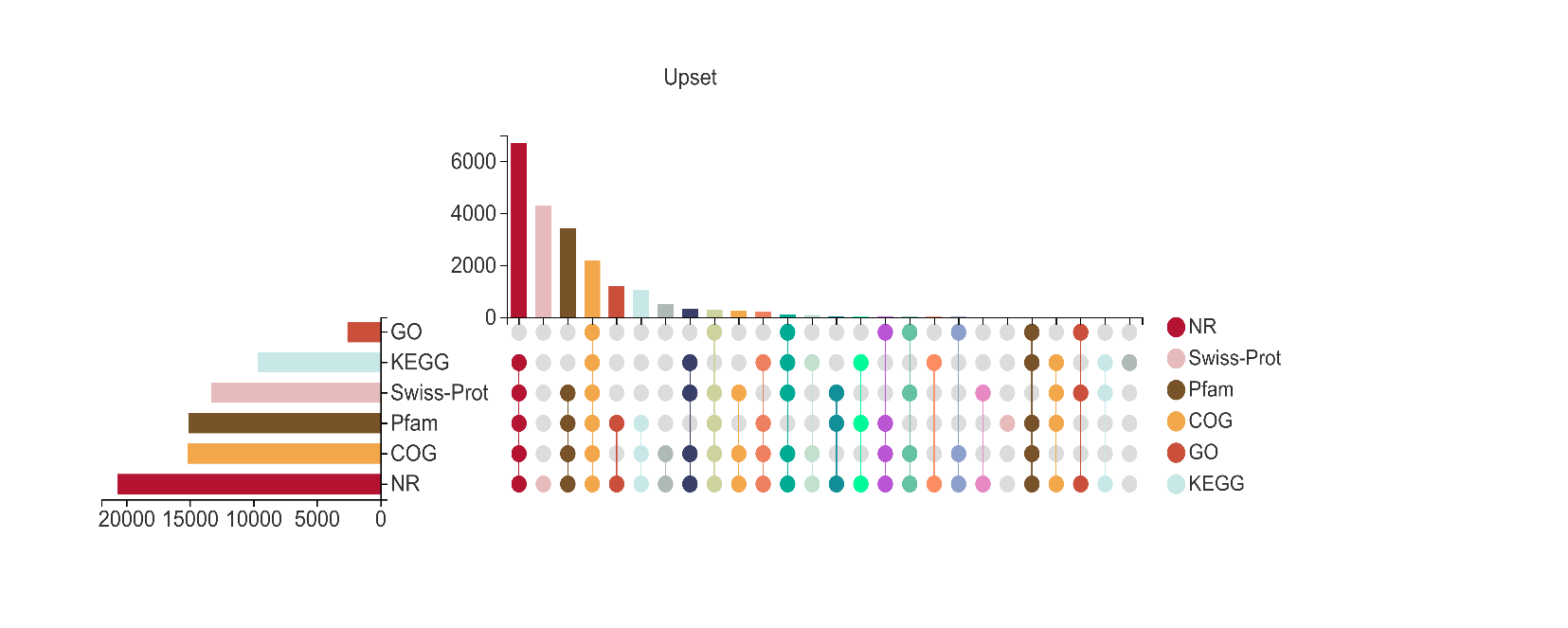


**Figure S14. Annotation of all mapped genes using the GO, KEGG, COG, NR, Swiss-Prot and Pfam databases**. The horizontal histogram on the left represents the statistical values of the elements of each set. Each point in the matrix represents a unique element of a set. The lines between the points represent unique intersections of different sets. The vertical histogram represents the corresponding values of the intersecting elements.
